# Structural and functional diversification in the teleost S100 family of calcium-binding proteins

**DOI:** 10.1186/1471-2148-8-48

**Published:** 2008-02-14

**Authors:** Andreas M Kraemer, Luis R Saraiva, Sigrun I Korsching

**Affiliations:** 1 Institute of Genetics, University of Cologne, Zuelpicher Strasse 47, 50674 Cologne, Germany

## Abstract

**Background:**

Among the EF-Hand calcium-binding proteins the subgroup of S100 proteins constitute a large family with numerous and diverse functions in calcium-mediated signaling. The evolutionary origin of this family is still uncertain and most studies have examined mammalian family members.

**Results:**

We have performed an extensive search in several teleost genomes to establish the *s100 *gene family in fish. We report that the teleost S100 repertoire comprises fourteen different subfamilies which show remarkable similarity across six divergent teleost species. Individual species feature distinctive subsets of thirteen to fourteen genes that result from local gene duplications and gene losses. Eight of the fourteen S100 subfamilies are unique for teleosts, while six are shared with mammalian species and three of those even with cartilaginous fish. Several S100 family members are found in jawless fish already, but none of them are clear orthologs of cartilaginous or bony fish *s100 *genes. All teleost *s100 *genes show the expected structural features and are subject to strong negative selection. Many aspects of the genomic arrangement and location of mammalian *s100 *genes are retained in the teleost *s100 *gene family, including a completely conserved intron/exon border between the two EF hands. Zebrafish *s100 *genes exhibit highly specific and characteristic expression patterns, showing both redundancy and divergence in their cellular expression. In larval tissue expression is often restricted to specific cell types like keratinocytes, hair cells, ionocytes and olfactory receptor neurons as demonstrated by *in situ *hybridization.

**Conclusion:**

The origin of the S100 family predates at least the segregation of jawed from jawless fish and some extant family members predate the divergence of bony from cartilaginous fish. Despite a complex pattern of gene gains and losses the total repertoire size is remarkably constant between species. On the expression level the teleost S100 proteins can serve as precise markers for several different cell types. At least some of their functions may be related to those of their counterparts in mammals. Accordingly, our findings provide an excellent basis for future studies of the functions and interaction partners of *s100 *genes and finally their role in diseases, using the zebrafish as a model organism.

## Background

In mammals the family of S100 calcium binding proteins has been a target of intensive study for over 40 years, since the first two family members were purified from bovine brain. Until today at least 20 members were described in humans and a growing number in several other mammalian species. They share an average identity of 40–60% on the amino acid level, but key features are more highly conserved. The *s100 *genes code for small, cytoplasmic or secreted proteins that exhibit a common structure consisting of two calcium-binding loops each flanked by two alpha-helices [1]. The N-terminal domain is a low affinity S100-specific domain, whereas the other one is a classical EF-Hand. A variable hinge region connects both domains. Structural examination revealed a hydrophobic region being exposed upon calcium binding which is thought to interact with hydrophobic regions of the target proteins. The calcium-binding domains also exhibit affinity for other divalent cations such as copper and zinc [2]. Some family members possess dysfunctional calcium-binding domains, but there is also evidence of calcium-independent interactions in this family (for review see [3-7]). For human S100 family members more than 90 potential target proteins, both intra- and extracellular ones, have been reported. Among the interacting partners are other calcium-binding proteins (several members of the annexin family), enzymes (e.g. aldolase A/C), cytoskeletal components (actin, tubulin), cell cycle regulator genes (p53), second messenger-synthesizing enzymes (adenylate and guanylate cyclase) as well as kinases. Accordingly, S100 proteins take part in many cellular processes which may be divided into five major groups: a) modulation of the activity of some protein kinases b) modulation of other enzymatic activities c) maintenance of cell shape and motility d) modulation of signal transduction pathways and e) regulation of calcium homoeostasis [8]. The active forms of S100 proteins often consist of homo- or heterodimers and even tetramers [9] or higher aggregates [10]. The expression pattern of the S100 family exhibits a remarkable degree of cell and tissue-specificity, so that many tissues and cell types have their unique protein composition and expression level [11-13].

There is no evidence for S100 proteins in invertebrates, however, the evolutionary origin of the family has not been studied much [14,15]. By far most publications deal with the mammalian family members, and in fact no systematic study has been performed in teleosts. With an intent to infer properties of the family in the most recent common ancestor of teleosts and tetrapods we have characterized the S100 family both in shark and lamprey, and in teleosts. We identified in each teleost species thirteen to fourteen *s100 *genes, which are under strong negative selection. Several of these genes appear to be considerably more ancient than the teleost speciation events taken into account here. Several mammalian *s100 *genes were found to have counterparts in teleosts, but eight novel genes restricted to teleosts were also detected. Furthermore we have investigated the expression of ten *Danio rerio s100 *genes by RT-PCR and *in situ *hybridization and report highly distinctive and specialized expression patterns, suggestive of similarly specialized functions of the different family members.

## Results

### The repertoire size of the S100 family of thirteen to fourteen genes is remarkably conserved across six divergent teleost species

The S100 proteins constitute a small family of calcium-binding proteins with highly specific expression patterns in mammals. So far the evolution of this family has not been studied in teleosts. Up to date only two *s100 *genes were reported in fish: a homologue of the mammalian S100A was found in loach and trout [16,17] and a new family member, ictacalcin, is present in several fish species (catfish, pufferfish, loach, trout and zebrafish), but absent in mammals [18-20]. These observations are consistent with the notion that the S100 family may be already well established in teleost fish, but may also diverge considerably from the mammalian repertoire. We therefore performed extended searches in the genomes of five different teleost species to establish the complete S100 repertoire in fish.

**Figure 1:**
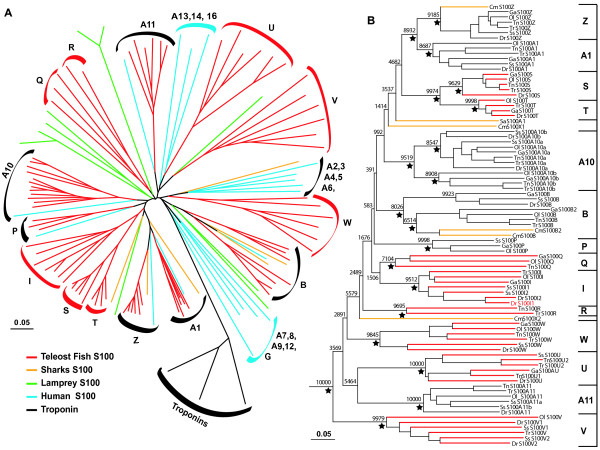
**Phylogenetic tree of the teleost and human *s100 *gene family**. A) 85 fish S100 (bony fishes in red and cartilaginous fishes in orange), 10 lamprey S100 (light-green) with human *s100 *genes (full set, excluding close relatives [38], light blue) and several representatives of another EF hand family, the troponins as an outgroup (black). B) Fish *s100 *genes from six teleost species and two cartilaginous fish (*Squalus acanthias and Callorhinchus milii*) are depicted. Catfish S100I [18] groups with zebrafish S100I (not shown). The Tn_S100T fragment (2nd exon, see Additional File 4) is not included for technical reasons. The colored names indicate fish *s100 *genes previously published. Stars indicate that the clades downstream of the node are supported by all three methods used for the phylogenetic analysis (NJ, ML and MP). The trees presented were constructed using the NJ method. Bootstrap support (total 10000 replications) is indicated at the major nodes. Scale bar indicates the number of amino acid substitution per site.

A recursive search strategy in the NCBI and Ensembl databases using the complete repertoire of human *s100 *genes as reference set, in combination with retrieval of automated ortholog predictions (see Methods) uncovered a total of 97 fish *s100 *genes. Fourteen *s100 *genes each were identified for zebrafish and *Takifugu rubripes*, and thirteen genes each for three-spined stickleback, medaka, and *Tetraodon nigroviridis*. In addition, fourteen *s100 *genes were found in EST libraries of another teleost, *Salmo salar*. All of these genes are novel, with the exception of one zebrafish and one *Takifugu *gene (see Figure 1). We believe that the result of the data mining approach used reflects the total repertoire of the *s100 *genes in the cases of *Danio rerio*, *Gasterosteus aculeatus, Oryzias latipes, Tetraodon nigroviridis *and *Takifugu rubripes*. In the case of *Salmo salar *several transcripts were found for each gene predicted, so that the repertoire reported here may approximate the complete S100 family present in this species.

Because novel fish-specific S100 subclades were found, it is necessary to expand the existing S100 nomenclature to include these new genes (Figure 1). We suggest the following criteria (in order of importance):

1. If any ortholog of the gene was described previously, that name is kept. This is the case for A1, A10, A11, B, P and Z members of the S100 family, which have mammalian orthologs, and for the fish-specific S100I.

2. Whenever new closely related genes correspond to a single named ortholog, that name is used, together with an additional numeral or letter to distinguish the fish genes from each other. This is the case e.g. for A10a and A10b of the S100 family.

3. Novel fish-specific genes and subfamilies are named consecutively by so far unused letters, starting with the letter Q onwards.

### S100 family size in cartilaginous and even jawless fish may be similar to that of bony fish

We have searched the available databases for S100 family members in cartilaginous fish (*Callorhinchus milii *and *Squalus acanthias*) and a jawless fish, *Petromyzon marinus*. We report ten different S100 family members in *Petromyzon marinus *and five *s100 *genes in *Callorhinchus milii *plus one gene in *Squalus acanthias *(Figure 1 and Additional File 1). All genes are novel, except three genes from *Petromyzon*[14], see also Additional File 2. The reported repertoire is expected to be incomplete, especially in the case of *Callorhinchus milii*, for which only 1.4 fold genomic coverage is currently available, and of course *Squalus acanthias*. Thus the total size of the cartilaginous and jawless fish S100 repertoires may be rather similar to that of teleost fish, despite marked sequence divergence. The latter necessitated further rules for nomenclature:

4. If a gene from a basal taxon is clearly related to two named genes of more derived species, we suggest to always use the preceding letter.

5. If no clear orthologs to any named gene can be delineated, the genes are named provisionally with x1, x2 etc. not to foreclose a coherent nomenclature for *s100 *genes from basal taxa, which will only be possible after completion of the respective genome projects.

### S100 is an ancient family with a complex pattern of gene gains and losses

In order to better understand the relationships and evolutionary history of the S100 family members we performed a phylogenetic analysis, using the Neighbor-Joining method [21], as well as Maximum Parsimony and Maximum Likelihood analyses using the PHYLIP package [22] (for details see Methods). Full length coding sequences of all teleost S100 genes were analyzed together with human S100 genes, using troponins, calmodulins and other related calcium binding protein with EF-Hand motifs [23,24] as outgroup (Figure 1, Additional File 1 and data not shown). This phylogenetic analysis shows twenty-seven different groups of orthologs, i.e. 27 different genes as the combined repertoire analyzed here (Figure 1A). Six of these genes are organized in closely related gene pairs (*s100a1/z*, *s100s/t, s100a10a/b*). The delineation of the *s100 *gene family, the identification of ortholog groups and the association in gene pairs were supported by all three tree-building algorithms used (Figure 1, Additional File 1).

Nearly a third of the family members are present only in fish – S100I and seven novel proteins, S100Q, S100R, S100S, S100T, S100U, S100V, and S100W.

Remarkably, very few of these fish-specific genes have the full set of five orthologs out of five fish genomes analyzed. When salmon is taken into account, only a single gene, *s100w*, is found in all 6 teleost species. In four other genes one ortholog is missing, the *Tetraodon nigroviridis *gene in *s100i*, the *Oryzias latipes *ortholog in *s100u*, and the *s100s, s100t *genes in *Salmo salar*. Only four and three species have representatives of the *s100v *and *s100q *genes, respectively. Finally, subfamily S100R is only found in pufferfish (Figure 1). On the other hand, restricted duplication events have occurred for S100I, S100V (zebrafish, salmon) and S100 U (pufferfish).

**Figure 2:**
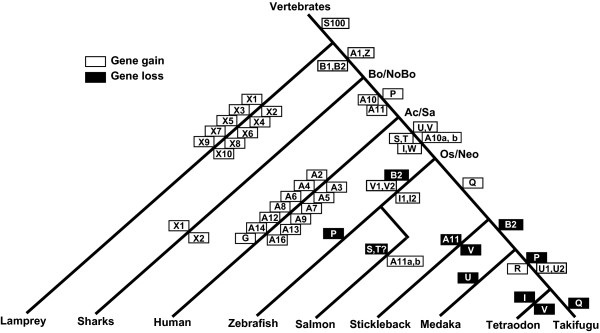
**Estimated minimal evolutionary age of S100 family members**. Open rectangles represent the gene gain events in each lineage, and black boxes represent the gene loss events. Inside each box is the name of the respective gene(s). A duplication event leading to a gene pair is indicated by a common rectangle. The major phylogenetic transitions are indicated: bo/nobo, bony fish/cartilaginous fish; ac/sa, actinopterygian/sarcopterygian split; os/neo, ostariophysii/neoteleostei segregation. The maximum parsimony principle was followed for construction of this scheme, i.e. minimal gene losses and gene gains were assumed. Thus gene gains are depicted at the last possible stage before additional gains would become necessary for explanation, but may in fact have occurred earlier, accompanied by gene loss in some lineages.

All teleost-restricted genes that contain both a zebrafish and another teleost representative (S100I, S, T, U, V, W) should have been present at least in the most recent common ancestor (MRCA) of *Neoteleostei *and *Ostariophysii *(zebrafish lineage diverged early from the more modern neoteleosts, to which the other four species analyzed here belong, salmon takes an intermediate position, closer to the *Neoteleostei *than zebrafish). Thus, the partial absence of several genes, e.g. S100I in pufferfish (*cf*. Figure 1) suggests a partial loss of these genes in the pufferfish family (*cf*. Figure 2) although it cannot be ruled out with certainty that some apparent gene losses are actually caused by inadequacies of the currently available databases. Other examples for restricted loss in some teleost species concern S100Q, U, V and the gene pair S100S/T (Figure 2, *cf*. Figure 1B).

Two of these teleost-specific *s100 *genes (S100Q, R) were not detected in the zebrafish genome despite extensive searches. The most parsimonious explanation therefore is an absence of these genes in the MRCA of *Neoteleostei *and *Ostariophysii, i.e*. a genesis of these genes in the *Neoteleostei *lineage (*cf*. Figure 2).

Six other genes are shared by teleost fish and mammalian species (S100A1, S100A10, S100A11, S100B, S100P and S100Z). Thus, all six genes should be more ancient than the split in *Actinopterygii *and *Sarcopterygii*, the separate teleost and tetrapod lineages. Four genes (S100A1, A10, B and Z) are highly conserved among all six teleost species, with at least one ortholog per species being present. However, other genes, particularly S100P, exhibit the same partial representation in teleost species as described above for some fish-specific *s100 *genes. Thus these genes appear to have been lost in some teleost sublineages. A graphical representation of all these phylogenetic inferences is given in Figure 2.

Four of the six *s100 *genes found in the shark genome also belong to this group of genes common to teleosts and mammals (the remaining two exhibit no unambiguous orthologs). One is an S100Z ortholog, two are S100B orthologs, and one (S100A1) is situated at the root of the S100A1-S100Z clade (Figure 1B). Thus, genes *s100a1*, *s100*b, and *s100*z appear to have emerged before the divergence of cartilaginous fishes from bony fish. Since the currently available shark genome is bound to be incomplete, it may be expected that about half of the extant *s100 *genes already existed in the MRCA of cartilaginous and bony fish. Moreover, the phylogenetic position of all cartilaginous fish *s100 *genes deep within the teleost phylogenetic tree demonstrates that the S100 family was already well established before the segregation of bony and cartilaginous fish (*cf*. Figure 2). In fact, the S100 family even precedes the earlier split in jawed/jawless vertebrates, because already ten family members were found in lamprey (Figure 1, Additional Files 1, 2). However, with possible exception of two genes related to S100B and one related to S100V, no ortholog relationship is apparent between any of the *Petromyzon marinus s100 *genes and those from more derived species.

Thus the phylogenetic tree reflects a complex evolutionary pattern characterized by frequent gene losses as well as extended gains of either single genes or whole gene subfamilies. Similar changes in the gene repertoire caused by complex genomic reorganization and translocation events are reported for EF hand proteins in general [25].

### Moderate overall similarity but high degree of conservation of motifs that are characteristic for the S100 family

**Figure 3:**
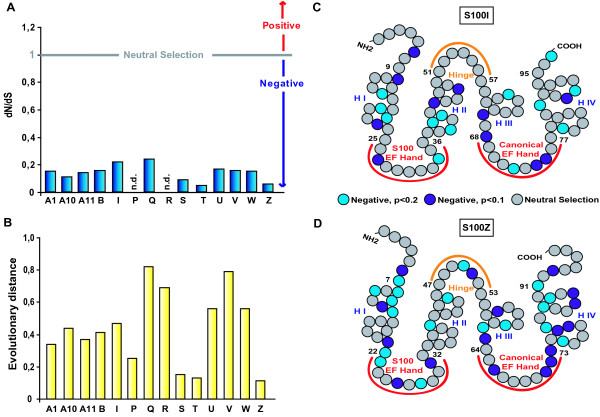
**Evolutionary distances and selective pressure on *s100 *genes**. A) dN/dS ratios of the S100 ortholog groups in which this analysis was possible (>2 genes/group), n.d., not determined. B) Amino-acid sequence average evolutionary distances within S100 ortholog groups. C, D) A representation of site-by-site selective pressure is shown on S100 sequences. The analysis is based on the nucleotide alignment of each ortholog group. SLAC analysis shows the probability of sites being under selective pressure (negative selection in light-blue (p < 0.2) or blue (p < 0.1), neutral selection in gray. Positive selection was not observed. Orthologs genes of the following species were included in this analysis: *Danio rerio, Gasterosteus aculeatus, Oryzias latipes, Tetraodon nigroviridis and Takifugu rubripes*; the results for two genes are shown. C) S100I, D) S100Z.

**Figure 4:**
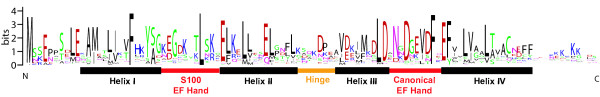
**Amino acid sequence conservation in the fish *s100 *gene repertoire**. Conservation is displayed as a sequence logo. In this representation, the relative frequency with which an amino acid appears at a given position is reflected by the height of its one-letter amino acid code in the logo, with the total height at a given position proportional to the level of sequence conservation. The regions corresponding to the Helices (H), the S100 EF Hand Calcium-binding domain, the Hinge and the Calcium-binding domain of the canonical EF Hand are numbered and indicated. Sequence alignments were manually edited (for details see Methods section).

The evolutionary distances between ortholog amino acid sequences range between 0.11 and 0.82 (Figure 3B, Additional File 3), whereas paralog distances range between 0.97 and 1.18 (Additional File 3). Because of this high heterogeneity among the S100 family and to obtain a second line of evidence in order to classify these genes reliably as members of the S100 family, we next examined the degree of conservation of the fish and mammalian genes. For that purpose, sequence logos from the amino acid alignments of either the fish or mammalian S100 proteins alone and of a combination of all the S100 proteins were created and used for a comparative analysis (Figure 4 and data not shown). All three sequence logos were very similar and showed the same general conservation pattern. The previously described typical structure of the mammalian S100 proteins formed by four helices (Helix I-IV) separated by a S100 EF hand calcium-binding domain, a hinge and a canonical EF hand calcium-binding domain [1] is conserved among the fish S100 proteins. A high number of conserved residues and signature motifs in defined positions of the sequence were identified in the teleost S100 proteins (Figure 4) and found to be conserved also among fish and tetrapods, consistent with the origin of the S100 family well before the *actinopterygian/sarcopterygian *split (Figure 2). These residues are located mainly in the helix domains and the calcium-binding regions, especially in the canonical EF hand. This result is in accordance with a recent study that classifies this region as phylogenetically older than the S100 EF hand domains [15]. In contrast, the hinge region and the C-terminus seem to be regions where there is more space for sequence variability (Figure 4). This is in accordance with the observation in mammalian S100 proteins that the selectivity in target binding is mainly assured by the hinge region and the C-terminal tail [2,7], i.e. in these regions a larger divergence is expected at least between paralogs.

Among the highly conserved amino acids are, as expected, the calcium-coordinating residues of the EF hands, *cf*. [1] and several hydrophobic residues involved in dimerization (helices I and IV) and binding to target proteins (helices III and IV) [26,27]. A conserved cysteine close to the end of helix IV (Figure 4, Additional File 4) has been shown in mammalian S100A10 and A11 to stabilize the dimer by intermolecular disulfide bridge formation [26,27]. This cysteine is part of a conserved hydrophobic pattern F--L---L---C---F of helix IV (hyphens indicate spacing, *cf*. Figure 4), which has the proper spacing to form the contact surface for dimerization. Similar conserved patterns of 3 to 5 amino acids are observed for the other three helices (L--A---L---F---S/A, helix I; L---L---L---L, helix II; V---M--L, helix III, *cf*. Figure 4 and Additional File 4).

Most EF hands found in the teleost genes appear to be functional as judged by sequence comparison, with exception of three proteins, S100R, U, V, and some neoteleost members of the S100B subfamily. The S100R protein has lost key residues of the canonical calcium-binding motif in the second EF hand loop, despite a generally good conservation in both domains, similar to the human S100A10 and A14 proteins [1]. The S100V protein has lost most key residues of the canonical motif [1].

In four of the five neoteleost S100B proteins a common three amino acid deletion has removed a key residue of the S100-specific calcium-binding loop. Nearly the same deletion also occurs in the S100V and the S100U proteins, as well as in the human S100A7, A10, the medaka S100A10a, and the shark CmS100B2, x2 (Additional File 4). The frequency of this deletion suggests a specialized function for such genes, which might act either as competitive antagonists, or alternatively, may adopt the calcium-bound conformation constitutively. Indeed, the latter has been demonstrated for the human A10 protein [26,28]. S100 genes with such deletions do not segregate together in the phylogenetic tree (Figure 1, Additional File 1), and the deletions vary slightly in length (2–4 amino acids) and position (2 to 4 amino acids left, right, or central to the conserved G65 in the S100 EF hand, Additional File 4). Thus, this kind of mutation has arisen several times independently. Some of these deletion events must have occurred in the MRCA of cartilaginous and bony fish (S100B), others appear to have originated in the MRCA of teleosts (S100U, V), and some are evolutionary late events restricted to a single species (Ol_S100A10a). In other words, this particular deletion may be considered a local optimum in the state space of S100 sequences, consistent with a particular function for these genes.

### Strong negative selection for s100 genes, but virtual absence of positive selection

As previously mentioned, paralog evolutionary distance at the amino acid level is very high, with an average value of 1.08, and a range from 0.97–1.18 (Additional File 3). Such high values pinpoint the high variability in the amino acid sequences of these genes found at the intraspecies level. In contrast, the average ortholog evolutionary distance value of 0.44 (with values ranging from 0.11–0.82) is about 2.5 fold lower than the one observed for the paralog comparisons (Figure 3B, and Additional File 3).

Prompted by both the high degree of intraspecies variability and the high degree of interspecies conservation of *s100 *genes we decided to investigate the evolutionary constraints that are acting on this gene family. A strong constraint is equivalent to strong negative selection. The degree of selective pressure is measured by investigating the relative frequency of non-synonymous (dN) *vs *synonymous (dS) substitutions [29]. When the number of dN equals the number of dS, the dN/dS ratio equals 1, which corresponds to neutral selection. If the number of non-synonymous changes is higher than the number of synonymous changes, then dN/dS >1, which indicates positive selection. On the other hand, if the number of synonymous (dS) changes is higher than the number of nonsynonymous changes, then dN/dS <1 and we are in the presence of negative selection [29]. Whenever possible, the overall dN/dS ratio within each ortholog group was determined. All the groups show very small values, ranging from 0.05 to 0.24, with an average global dN/dS value of 0.14 (Figure 3A), which is evidence for strong negative selection.

These low dN/dS values combined with the high divergence between the S100 genes suggest not only an ancient origin of these genes but also indicate that individual genes of this family are slowly evolving.

However, as the strong negative selection could be masking some positive selection events at the individual codon level, we also determined the dN/dS ratio separately for each individual sequence position. Results are shown for two of the ortholog groups, the S100I and S100Z. These two clades represent two different evolutionary scenarios, the first showing evidence for recent gene duplications in zebrafish and lacking one ortholog in *Takifugu rubripes*, while the second is a very well conserved group with orthologs in all six fish species. Also they show two different values for their evolutionary distances, of 0.47 and 0.11 respectively (see Figure 3B and Additional File 3). As predicted by the global dN/dS analysis, a considerable portion of the total sequence – 25% for the S100I and 35% for S100Z – is under negative selection, with the second value being closer to the average value of 31 sites per ortholog group (Figure 3B and 3C and Additional File 5). No evidence for positive selection was found, suggesting that positive selection does not appear to play a significant role in the evolution of the *s100 *genes.

A comparison between S100I and S100Z shows rough similarity in the pattern of negative selection (focused in the helices I and IV and the second calcium-binding domain), although no specific motifs could be identified between genes (Figure 3C, D).

### Gene structure of s100 genes is highly conserved between fish and mammalian species

**Figure 5:**
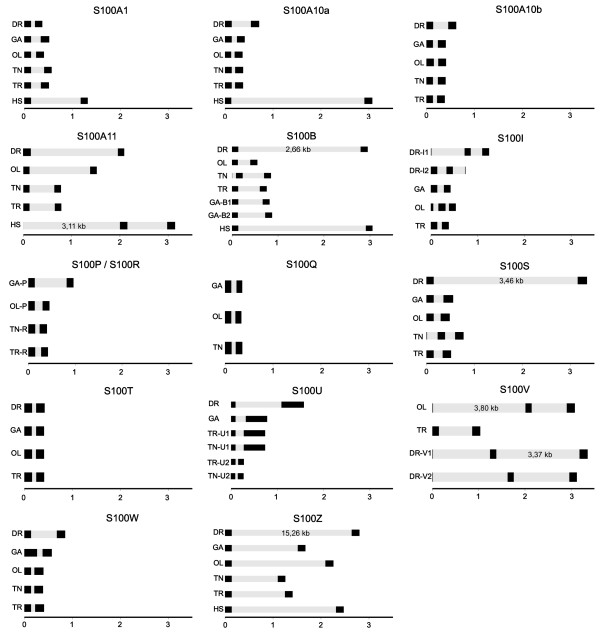
**Genomic structure of 66 teleost fish *s100 *genes**. Predicted exon/intron structure is drawn to scale (kb units) for all *s100 *genes with genomic sequence information: fourteen genes each for zebrafish (Dr; *Danio rerio*) and takifugu (Tr; *Takifugu rubripes*), thirteen genes each for medaka (Ol; *Oryzias latipes*) and stickleback (Ga; *Gasterosteus aculeatus*), twelve genes for tetraodon (Tn; *Tetraodon nigroviridis*). Exons are represented by black filled rectangles and introns are represented by the gray boxes connecting the exons. Where present, the human ortholog is drawn for comparison (Hs, *Homo sapiens*).

The mammalian *s100 *genes are found to have a common structure consisting of usually three exons [5]. The first exon contains only 5'UTR, part of the second and third exons code for the ORF and a 3'UTR is located in the last portion of the third exon [24]. For the fish *s100 *genes we restricted analysis to the coding exons, since prediction of noncoding UTRs is currently not very reliable. We found that 59 of the analyzed 66 teleost *s100 *genes conform to the pattern observed in ortholog genes in humans. However, another seven teleost fish genes have their ORF split in three exons, one of them in most cases very small and always located at one end of the ORF (Figure 5). For zebrafish several, but not all, of these predicted exons have been confirmed by RT-PCR (*cf*. Additional File 6).

Each major exon contains one complete EF-hand domain. Without exception these two exons are joined at the same position in the sequence (Additional File 4). Thus these exons may be as old as the S100 family itself and the first such gene may have arisen from a duplication of this domain. In contrast, the intron/exon borders of the small exons (Additional File 4) show a variable position in the N-terminal and C-terminal regions and occur in few species only, consistent with a late origin in the teleost lineage.

### S100 fish genes are distributed both as singletons and small clusters in the genome

**Figure 6:**
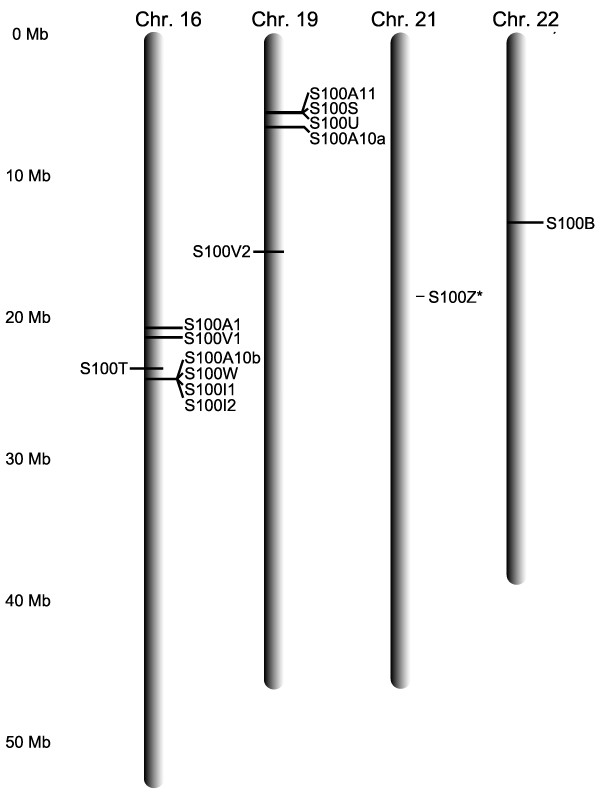
**Chromosomal location of the *s100 *genes in zebrafish**. Genes are distributed in two clusters and three singletons on four different chromosomes. Vertical scale, distance in Mb. Orientation of the genes is indicated by left/right position (reverse/forward strand, respectively). Genomic location was retrieved from the Ensembl database, release 47, based on assembly zv7, April 2007 [50], see also Additional File 2). The genomic location of *s100z *is currently unclear.

All mammalian *s100a *genes are clustered in the genome, whereas S100B, G, P, and Z are located as singletons on different chromosomes [1,30]. In zebrafish the scenario is somewhat similar, with two smaller clusters of 3.5 and 1.0 MB each located on the chromosomes 16 and 19, respectively, and composed of seven (S100A1, S100A10b, S100I.1, S100I.2, S100T, S100V1 and S100W) and four (S100A10a, S100A11, S100S and S100U) genes, respectively. Similarly, in the other teleost fish species analyzed small clusters of maximally five genes are found (*cf*. Additional File 2). These clusters always contain phylogenetically less related genes and, in contrast to the situation in mammals, cluster gene associations differ between species. However, and remarkably so, there are two *s100 *gene pairs, which are stably linked in the teleost genome, whenever present in the same species, albeit not closely related phylogenetically (S100A10a/S100I and S100A11/S100S, *cf*. Figure 6 and Additional File 2). Conversely, a closely related gene pair (S100A1/S100Z) consists of a singleton gene (Z) and a member of a cluster (A1). Thus, among teleost *s100 *genes, genomic linkage is not at all correlated with degree of phylogenetic relationship, consistent with an ancient evolutionary origin of these clusters.

In total, three zebrafish genes, S100B, S100V2 and S100Z, are present as singletons in the chromosomes 22, 19, and 21, respectively (Figure 6). This feature is conserved across all five teleost species studied; additionally S100P, which is absent in zebrafish, is always a singleton, whenever it occurs (*cf*. Additional File 2). The isolated genomic location of these family members appears to be an evolutionary ancient feature of the S100 family, since all members of this group also found in mammals (S100B, P, Z) share the singleton location.

The two clusters present in zebrafish are situated on chromosomes 16 and 19, which are largely syntenic [31] and result from the teleost-specific whole genome duplication, which occurred after the *actinopterygian/sarcopterygian *split [32]. Two of the three gene pairs observed in the teleost S100 repertoire (S/T, A10a/b) are distributed between these two clusters (Figure 6), consistent with a duplication of their ancestral genes by this whole genome duplication nearly 400 million years ago. This interpretation is supported by the observation that the gene pair A10a/b has a single mammalian ortholog. Moreover, very similar clusters are present in chromosomes 8 and 21 from *Tetraodon nigroviridis *and chromosomes 11 and 16 from *Oryzias latipes *(*cf*. Additional File 2). Both chromosome pairs are syntenic to each other, and to zebrafish chromosomes 16 and 19 [31,32]. The third recognizable gene pair in teleosts (A1/Z) is shared both with mammals and with cartilaginous fish, but A1 is always a member of a cluster (where determinable), whereas Z never is, consistent with a very ancient origin of this gene duplication, before the emergence of the cluster itself. In total, three genes from the human S100A cluster on chromosome 1, A1, A10 and A11 are found in the teleost clusters. Together with the synteny of this region of human chromosome 1 with the corresponding teleost cluster regions [31,32] these data are consistent with a small ancestral cluster of *s100 *genes in the MRCA of teleosts and tetrapods plus some isolated ancestral *s100 *genes.

In contrast, other gene pairs occur closely linked in the chromosome (e.g. the two *s100i *genes of zebrafish), indicating a local tandem duplication event as cause of such gene pairs.

### Expression patterns of s100 genes range from highly specific to fairly broad distributions

To obtain an overview of the tissue specificity of expression for the whole family we performed RT-PCR with twelve different tissues from adult zebrafish.

**Figure 7:**
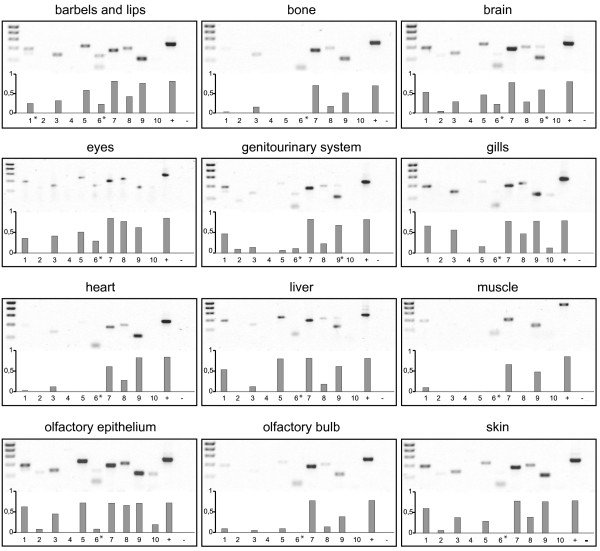
**Expression of ten *s100 *genes in adult zebrafish by RT-PCR**. mRNA was extracted from adult zebrafish tissue and transcribed into cDNA. Primer positions for PCR see Materials and Methods. Twelve different tissues were examined (barbels and lips, bone, brain, eyes, genitourinary tissue, gills, heart, liver, muscle, olfactory bulb, olfactory epithelium, skin). Band intensity was quantified to obtain an estimate of abundance to compare expression levels of several genes at least within the same tissue. Asterisks indicate bands at unexpected size (resulting from unspecific amplification) that were not quantified.

**Figure 8:**
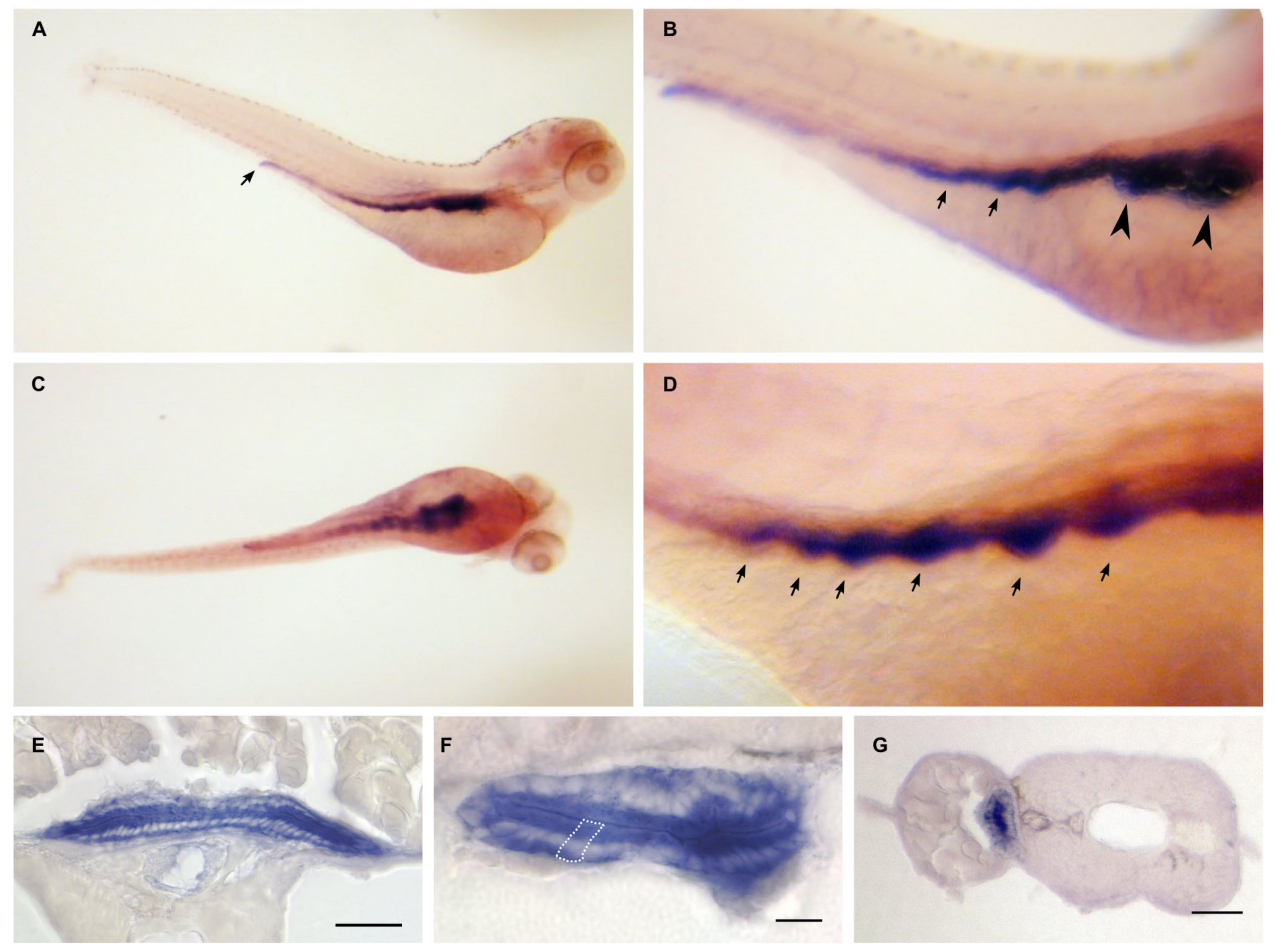
**Dr-S100A10a expression pattern by whole mount in situ hybridization**. Five day old zebrafish larvae were hybridized with RNA antisense probe. Panels A) to D), whole mounts; panels E) to F), sectioned after hybridization. Scale bars, 30 μm. A) Lateral view shows expression restricted to the whole intestinal tract including the anal region. B) Enlarged view, anterior is to the right, arrows point to the label in intestine. C) Ventral view, no other A10a-expressing regions are detected. D) Gut loops with high expression levels are pointed out by arrows. E, F) Cross sections of whole mount hybridizations at the level of the intestine. Only epithelial cells are strongly labeled, see white enclosure of a single epithelial cell in panel F). G) Gut epithelial cells are labeled as well.

**Figure 9:**
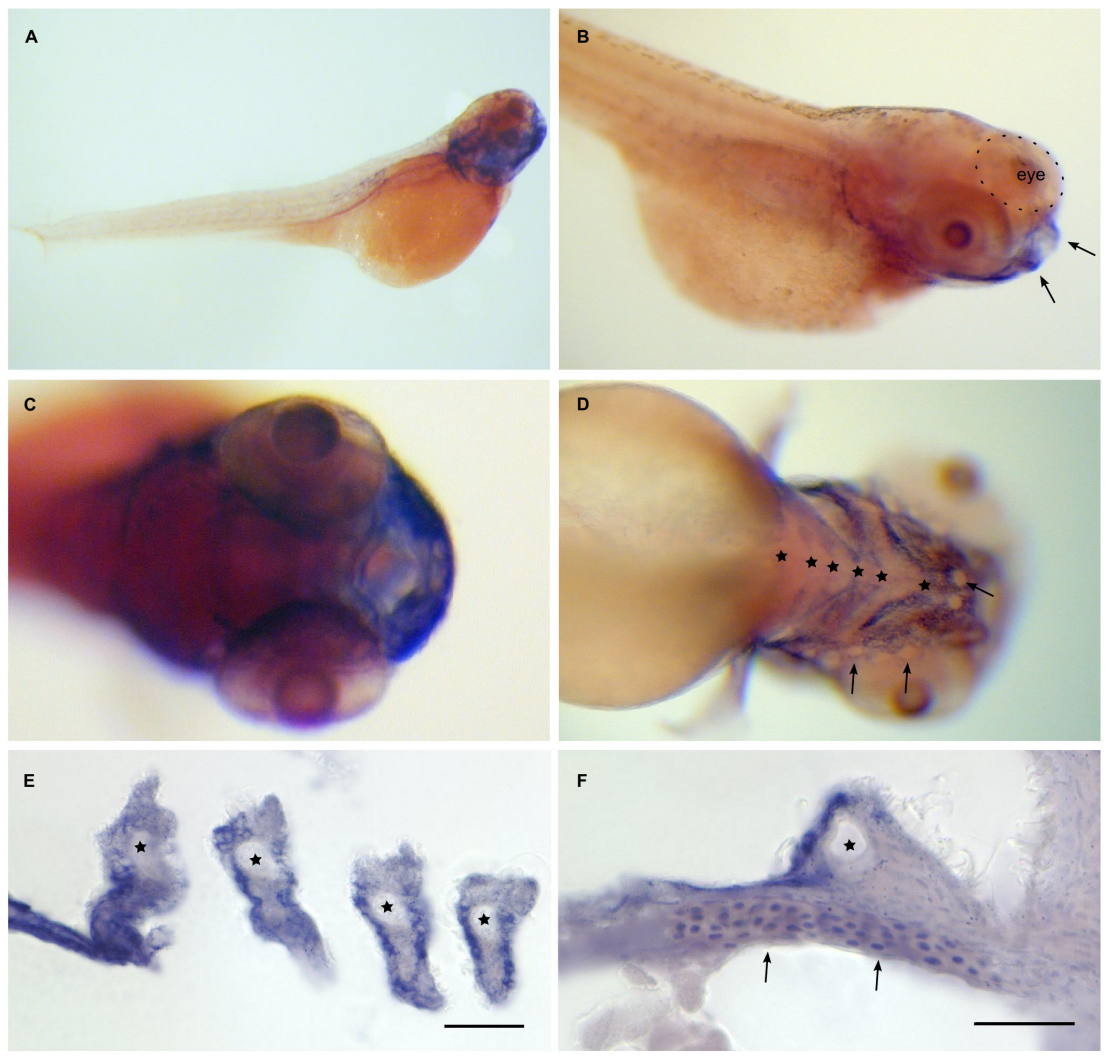
**Dr-S100A10b expression pattern by whole mount in situ hybridization**. Five day old zebrafish larvae were hybridized with RNA antisense probe. Panels A) to D), whole mounts; panels E) to F), sectioned after hybridization. Scale bars, 30 μm. A) Lateral view shows expression in the lower jaw. B) Lateral oblique view, lip region (arrows) expresses S100A10b. C) Frontal view (ventral to the right) shows expression in the mouth region. D) Ventral view (anterior is to the right), expression is visible in six branchial arches (asterisks), neuromasts (arrows) are not stained. E) Expression in branchial arches (asterisks) is limited to the epithelial layer. F) The seventh branchial arch (asterisk) is also expressing A10b, as well as cells of the pectoral fin (arrows).

**Figure 10:**
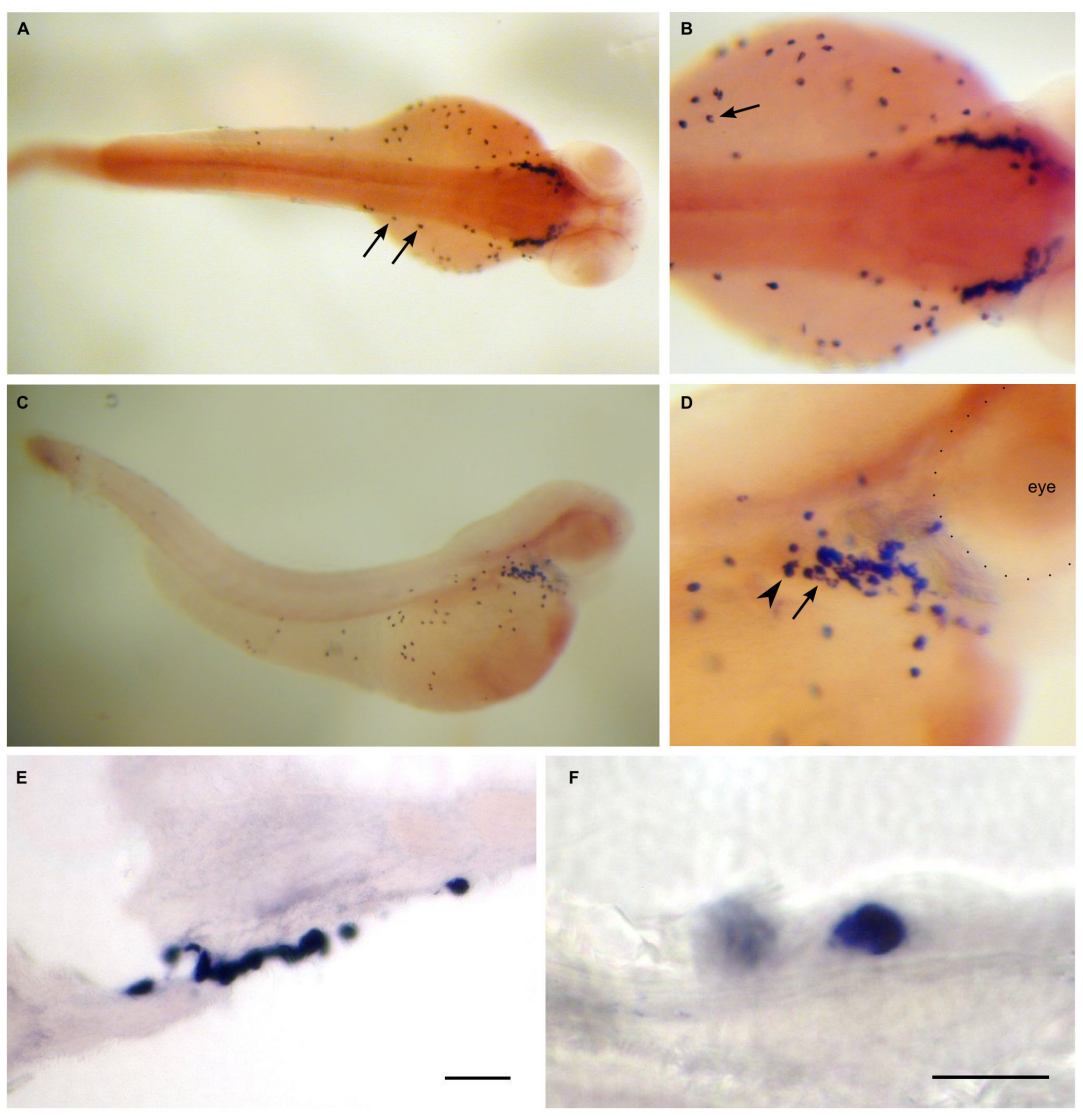
**Dr-S100A11 expression pattern by whole mount in situ hybridization**. Five day old zebrafish larvae were hybridized with RNA antisense probe. Panels A) to D), whole mounts; panels E) to F), sectioned after hybridization. A) Dorsal view, isolated large cells, mostly on the yolk sac, are labeled. B) Close up of A), an isolated cell with typical crescent-shaped soma signal is visible (arrow). C) Lateral view, a cluster of cells sits in the pericard, several cells are found on the yolk sac, a few in the skin. D) Close-up of the pericard region, expression intensity appears to vary (Arrowhead, arrow). E) Section through the cell cluster, scale bar 30 μm. F) Section with a single labeled cell, scale bar 10 μm.

**Figure 11:**
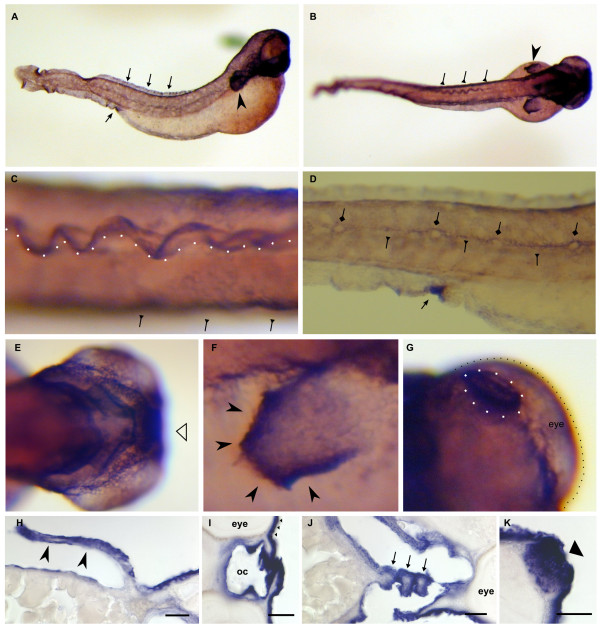
**Dr-S100I.1 expression pattern by whole mount in situ hybridization**. Five day old zebrafish larvae were hybridized with RNA antisense probe. Panels A) to G), whole mounts; panels H) to K), sectioned after hybridization. Scale bars 30 μm. A) Lateral view, strong ubiquitous expression is seen in the skin, the urogenital opening (bottom arrow), the rim of the dorsal fin (top row of arrows), the pectoral fin (arrowhead) and the lower jaw. B) Dorsal view, strong expression in the pectoral fin, the dorsal fin, and the lateral line (triangle-headed arrows) C) Enlargement of dorsal view, expression in the lateral line (arrows) and the dorsal fin (rim indicated by white spots). D) Larger magnification of urogenital opening (arrow) and the labeled lateral line (triangle-headed arrows) surrounding the neuromasts (diamond-headed arrows), which are not labeled. E) Ventral view, strong expression in branchial arches is seen. F) Enlargement of pectoral fin, especially the rim is heavily labeled. G) Dorsal view, expression in the olfactory placode (white dots). H) Cross section of the pectoral fin. I) Pharynx is intensely labeled. J) Three branchial arches (asterisks) are cross-sectioned; the mesenchyme including the cartilaginous bar is devoid of staining. K) The olfactory placode is heavily stained.

**Figure 12:**
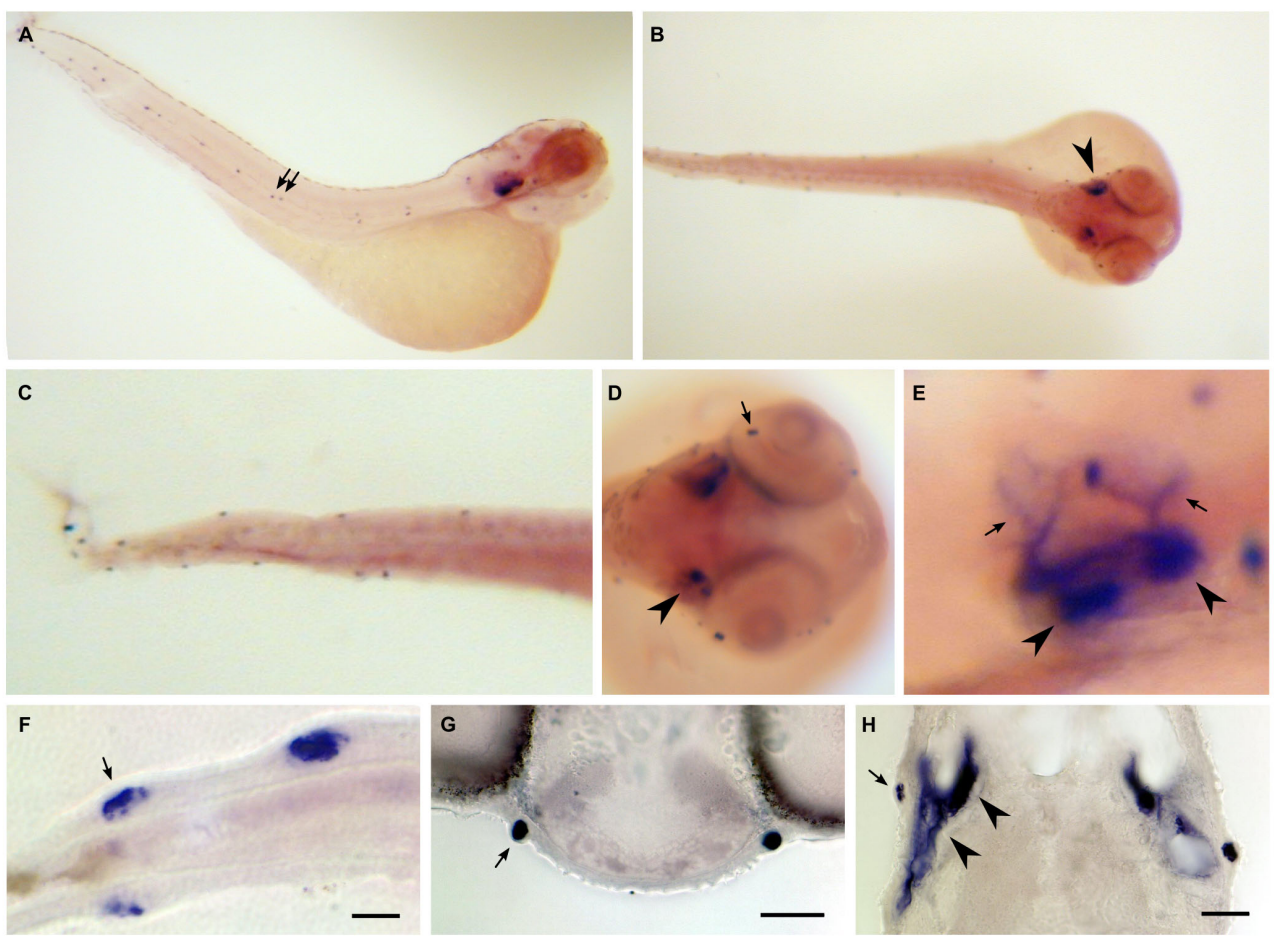
**Dr-S100S expression pattern by whole mount in situ hybridization**. Five day old zebrafish larvae were hybridized with RNA antisense probe. Panels A) to E), whole mounts; panels F) to H), sectioned after hybridization. A) Lateral view, expression in neuromasts (arrows) and otic placode is visible. B) Dorsal view, staining in both otic placodes (arrowhead) is seen. C) Magnification, neuromasts in trunk and tail are labeled. D) Frontal view of the head region, expression in neuromasts (arrow) and the otic placode (arrowhead) can be seen. E) Magnification of the ear region, expression in several spots (arrowheads) in the otic placode and in climbing fibers (arrows). F) Expression in tail neuromasts, all hair cells seem to be stained (arrow), the ring of hair cell nuclei is devoid of staining. Scale bar 10 μm. G) Expression in a symmetrical pair of neuromasts in the head region, scale bar 30 μm. H) Staining in the ear, anterior is up. Hair cells underlying the otoliths show strong expression (arrowheads). A neuromast (arrow) is also visible on each side. Scale bar 30 μm.

**Figure 13:**
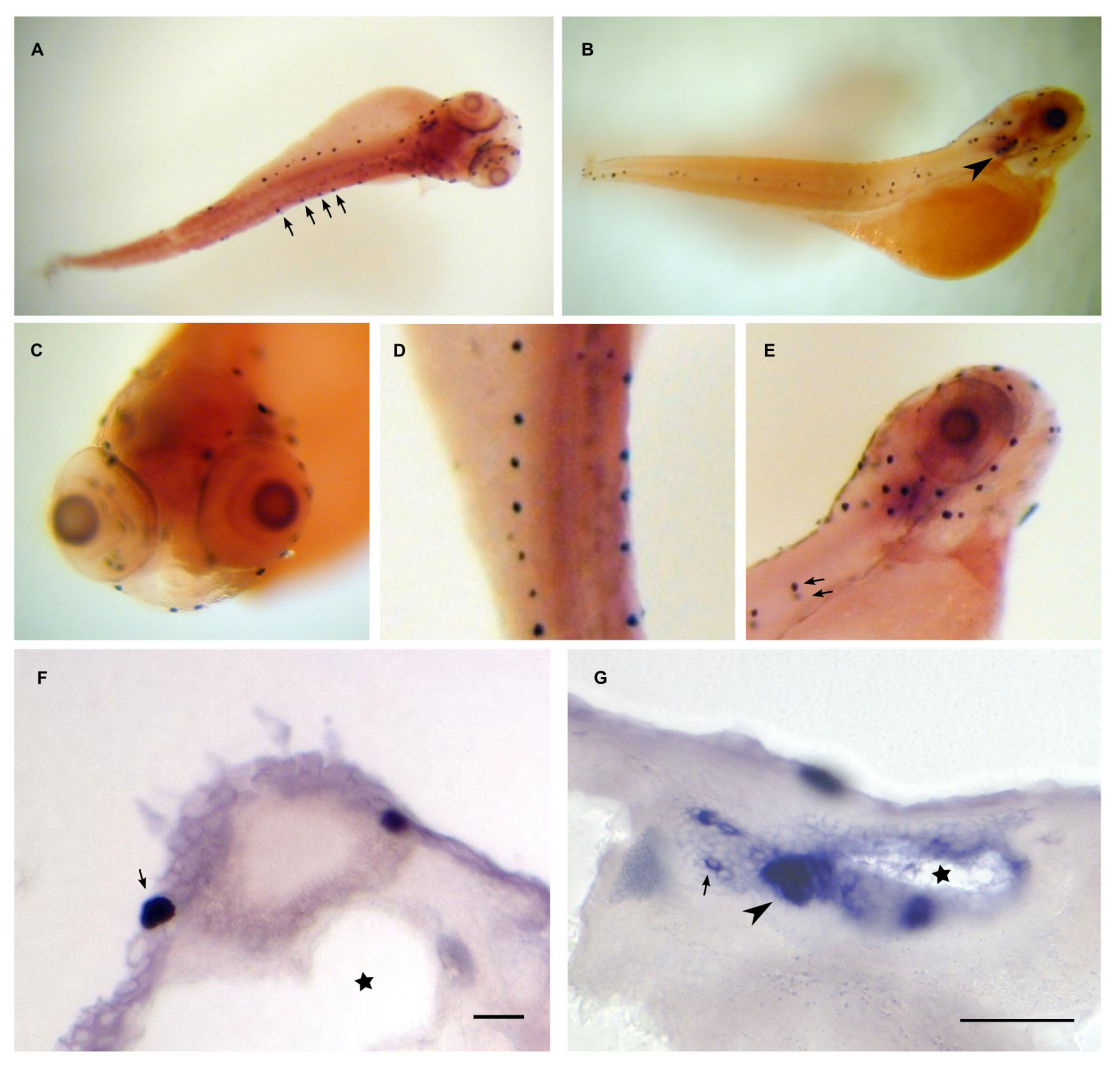
**Dr-S100T expression pattern by whole mount in situ hybridization**. Five day old zebrafish larvae were hybridized with RNA antisense probe. Panels A) to E), whole mounts; panels F) to G), sectioned after hybridization. Scale bars 30 μm. Dorsal (A) and lateral (B) view show expression in lateral line neuromasts (arrows) and the otic placode (arrowhead). C) Frontal view of the head region. Several labeled neuromasts are visible. D) Magnification of the trunk region from panel A), view from dorsal. E) Enlarged lateral view of the head region, over ten labeled neuromasts are seen. One neuromast and its contra lateral counterpart are indicated by arrows.

**Figure 14:**
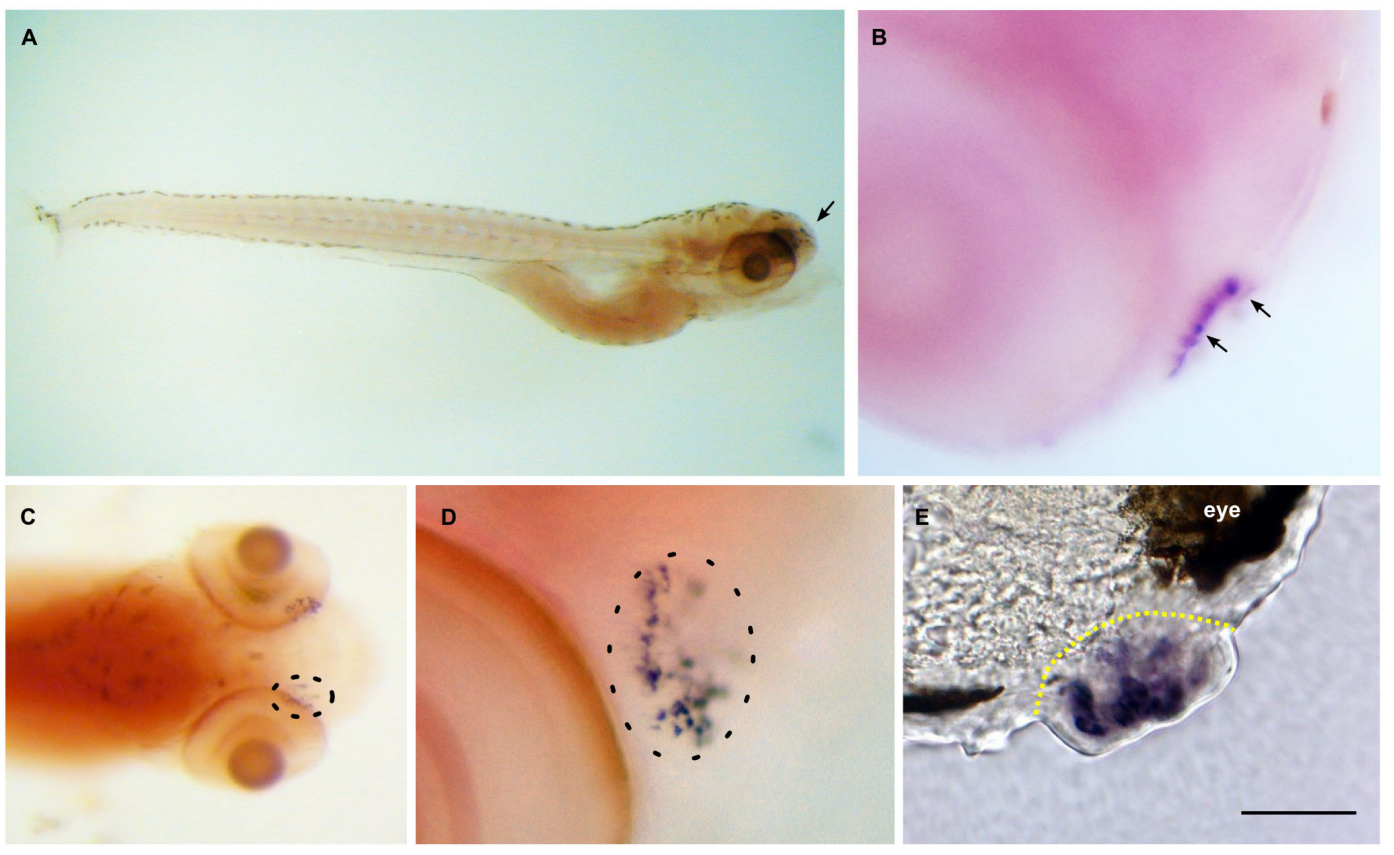
**Dr-S100Z expression pattern by whole mount in situ hybridization**. Five day old zebrafish larvae were hybridized with RNA antisense probe. Panels A) to D), whole mounts; panel E), sectioned after hybridization. Scale bar 30 μm. Lateral view. Expression in the larva is restricted to the olfactory placode (arrow). B) Closer view of the olfactory placode shows several large, labeled cells (arrows). C) Dorsal view of the head region, labeled cells in both olfactory placodes are visible, right placode encircled with dashed line. D) Enlarged view of a single olfactory placode (circled). Labeled cells are a subpopulation within the olfactory placode. E) Cross-section through an olfactory placode (delineated by yellow dashed line), several labeled cells are visible.

For a higher spatial resolution of the expression patterns at the cellular level we performed whole mount *in situ *hybridization of zebrafish larvae 5 days post fertilization. At this stage zebrafish have completed organogenesis and major behavioral patterns are already functional. Eight of ten genes analyzed show expression in the larval stage, with expression patterns ranging from highly restricted to spatially broad distributions (Figs. 8, 9, 10, 11, 12, 13, 14). The results of the *in situ *hybridizations are often consistent with those from the RT-PCR (Figure 7). A general tendency of the RT-PCR to show broader expression may be related to the higher sensitivity of that method compared to *in situ *hybridization. Additionally, a developmental regulation, i.e. a late onset of expression, may explain some of the differences in the results, especially the absence of two S100 transcripts (A1 and B) in larval tissues (although technical reasons cannot be ruled out for A1). For S100B a late onset of expression was confirmed by the detection of strong signals in adult brain (data not shown).

### Highly restricted expression of S100 genes within the sensory nervous system

Three zebrafish *s100 *genes show a remarkably restricted expression in just one or two different larval neuronal populations.

Zebrafish S100S is expressed in the neuromasts of the lateral line and the inner ear (Figure 12A–H). Labeled structures in the inner ear may include hair cells (Fig 12E). In sections of the tail region the central portion of the neuromasts is labeled (Figure 12F), consistent with an expression in sensory hair cells. Thus in the larvae particular sensory structures and possibly some neurons innervating them are labeled. The RT-PCR results (Figure 7) show a general expression in all sensory systems examined which are in detail barbels and lips, brain, eyes, olfactory system and additionally the genitourinary system.

The *in situ *expression pattern of S100S is quite similar to that of the highly related zebrafish S100T, which is also expressed in the neuromasts of the lateral line and the inner ear (Fig 13A–G), the latter in lower levels compared to S100S. Zebrafish develop approximately 9–12 neuromasts along one side [33], which have their counterparts on the opposite side and all of them are stained by either probe. In RT-PCR S100T expression is additionally observed in non-sensory tissue such as bone, gills, heart, liver and muscles.

Zebrafish S100Z (Fig 14A–E) is the gene with the spatially most restricted expression pattern in the larvae. Only a single tissue expresses S100Z, the olfactory placode, and within that tissue expression is restricted to a few large cells (Fig 14B,D,E), by morphology presumably neurons. A far broader expression pattern in adult fish is suggested by the RT-PCR results and may suggest a late ontogenetic onset of expression in those tissues.

The related S100A1 was not detected at all in larval tissues, but shows an expression in the adult genitourinary system, skin, olfactory epithelium and brain according to RT-PCR-results.

### Non-neuronal expression of two s100 genes, restricted to a single cell type or tissue

Zebrafish S100A10a shows one of the most restricted patterns (Figure 8A–G). Expression is restricted to the intestinal bulb and the intestine with intestinal loops being clearly visible. Expression slightly decreases from intestinal bulb to comparatively low levels in the anal region. RT-PCR was negative, consistent with the described specificity (digestive tissue was not included there). The expression is limited to the epithelial cells of the intestine and gut (Figure 8E–G).

Zebrafish S100A11 exhibits an extremely specific *in situ *hybridization pattern (Fig 10A–F). S100A11 seems to be restricted to isolated large cells located on the yolk sac and the pericardial cavity of the fish. There are about 150 cells labeled in the whole larva. RT-PCR with adult tissues suggests expression in gills and olfactory epithelium. The cellular morphology, larval staining pattern and the adult expression in gills suggest expression in MR-cells, also known as ionocytes [34,35]. These cells control ionic composition of body fluids, especially calcium levels. Results obtained by RT-PCR indicate a much broader distribution, possibly at levels below the threshold for *in situ *hybridization.

### Three S100 genes exhibit broad expression in several tissues and cell types

Zebrafish S100I.1 (Ictacalcin) expression (Figure 11) was previously described by [18] and [36]. Our data are consistent with their findings of expression in the epithelial cells of barbels, gills, olfactory rosettes and skin. S100I.1 is expressed throughout the whole skin with high levels of expression in the rim of the dorsal (Fig 11C) and pectoral (Fig 11F, H) fin. High expression levels were observed in the urogenital opening (Fig 11D). Moreover, S100I.1 exhibits also strong expression in cells of the olfactory placode (Fig 11G,K). Additionally expression can be observed throughout the pharyngeal arches (Fig 11E,I,J) and the lateral line excluding the neuromasts (Fig 11D), which appear as light spots in the middle of the larvae. In this respect S100I.1 shows the complementary pattern to S100S, T, which label the neuromasts, but not the surrounding tissue.

The *in situ *hybridization signals may represent the combined expression pattern of S100I.1 and S100I.2, since their probes share over 90% homology at the nucleotide level, and thus cross-reactivity cannot be excluded. However, RT-PCR results for S100I.1 are expected to be specific, since a primer from its unique, short exon was used. RT-PCR results for S100I.2 probably represent a mix of both S100I transcripts, since no completely specific primers were possible there. The results show expression of both genes in all tissues tested, with exception of muscle, where only S100I.2 could be detected.

Zebrafish S100A10b (Fig 9A–F) exhibits high expression in the lower jaw (Figure 9A) in particular in the branchial arches. Epithelial cells are labeled, but cartilaginous tissue is devoid of staining. Olfactory placode and the whole larval trunk do not express S100A10b. Thus this expression pattern is a subset of that observed for S100I.1. Unexpectedly, RT-PCR reveals expression of S100A10b in every tissue examined.

### Partial correlation of expression pattern similarity with coding sequence similarity

The highly related genes S100S and S100T show a nearly identical larval expression pattern, and the duplicate S100I genes appear to exhibit a very similar expression pattern as well. However, expression patterns for the duplicate A10a genes are very different, with the A10b pattern a subset of the unrelated S100I.1, I.2, and a completely different pattern being observed for A10a. This partial correlation is consistent with the interpretation that in some cases (S100S, T) regulatory elements have been conserved alongside the coding regions of these genes, which are spread far apart in the genome. Additionally, for the two S100I genes, which are clustered, regulatory elements shared within the cluster might synchronize the expression pattern. In this context it is noteworthy that the close genomic linkage of S100I to S100A10b is conserved in the genome of all fish species that possess both genes, and indeed, A10b expression is quite similar to that of S100I (*cf*. Figs. 9, 11), consistent with partially shared regulatory elements. On the other hand, A11 and S are also linked closely, and their expression patterns are completely different. Thus regulation of *s100 *gene expression follows a complex pattern presumably involving several different mechanisms as suggested for humans, *cf*. [37].

## Discussion and Conclusion

The S100 family represents the largest family of calcium-binding proteins with EF hands. S100 proteins have multiple and essential functions in many different tissues, mainly in cells of epithelial origin. However, most studies have been performed in mammals, and the evolutionary emergence of the family is not clear. We have performed an extensive search in six teleost genomes and identified in total fourteen distinct subfamilies, which constitute the fish *s100 *gene repertoire. Individual species exhibit thirteen to fourteen different *s100 *genes. Upon closer investigation a complex pattern of gene gain and gene loss emerges, which results in a roughly stable total repertoire size for each species. A similar family size is observed in amphibians (13 genes for *Xenopus laevis*, S.I.K., *unpublished observation*), whereas a preponderance of gene gains leads to an enlarged S100 repertoire size in mammals (23 acknowledged genes in humans [38]).

The presence of several S100 family members from cartilaginous fish (shark) deep inside the phylogenetic tree implies that the S100 family predates at least the segregation of bony from cartilaginous fish. Moreover, already ten members of the S100 family have been identified in a jawless primitive fish (lamprey). Thus the origin of the family may lie with very early vertebrate stages although individual family members only become reliably recognizable in the time period between the jawed/jawless segregation and the divergence of cartilaginous from bony fish around 560 to 530 million years ago [39].

The somewhat variable composition of each species repertoire suggests that gene function may be variable between species. In fact, an A11-like gene from *Xenopus *[40] is expressed in olfactory receptor neurons, i.e. may have acquired the function served in zebrafish by S100Z. Such flexibility is observed irrespective of a generally strong negative selection within the individual *s100 *genes, i.e. the evolutionary changes within the regulatory regions may be much faster than those within the coding regions.

On the other hand, some functional specializations appear quite stable, which may be inferred from the expression of human S100A10 in polarized epithelia [11,28,41] parallel to the expression of the zebrafish S100A10a in the intestinal epithelium. However, it should be noted that human A10 is constitutionally in the, calcium-bound' configuration due to a deletion in the S100 EF hand, in contrast to the zebrafish A10 genes. Moreover, the other member of this gene pair (S100A10b) shows a radically different distribution. Another example is S100B, which is expressed in brain in zebrafish as well as in mammalian species [7,42].

The genomic structure of teleost *s100 *genes is markedly similar to that of mammalian genes, with two to three coding exons in similar distances, and a conserved domain structure of one EF hand per each main exon. Some similarities are also observed for the genomic location. Most mammalian *s100 *genes lie together with some other genes in one large cluster, the so-called epidermal differentiation complex (EDC) [43,44] for which common regulatory elements have been discussed (but see [37]). Two smaller clusters of four to seven members in teleosts are syntenic to the human cluster and thus suggest the presence of an ancestral small cluster of *s100 *genes. Teleost cluster configuration does not correlate with sequence similarity, in contrast to the mammalian EDC. Thus the teleost clusters may represent the ancestral cluster composition more closely, while extended local gene duplications appear to have enlarged the mammalian cluster.

While the predominantly epithelial and epithelial-derived cellular expression of mammalian *s100 *genes is maintained in zebrafish, the actual regulatory mechanisms responsible for this similar expression may be different. No evidence for shared regulatory elements is observed in teleost fish, as judged from both sequence divergence and expression pattern similarity of neighboring genes (with the possible exception of A10b and I1).

Zebrafish *s100 *genes exhibit highly specific and characteristically different patterns of expression like their mammalian counterparts (*cf*. [11-13]). Duplicated genes show both redundancy and divergence in their expression pattern. Larval expression is limited to different types of epithelial cells and neural cells of epithelial origin (olfactory receptor neurons, hair cells and ionocytes). Our findings provide a firm basis for unraveling the molecular nature of the S100-like immunoreactivity observed in teleost fish tissues in several earlier publications, e.g. [45-47].

Taken together we have identified and characterized the teleost *s100 *gene family with respect to gene structure and expression pattern. These genes exhibit characteristics similar to the mammalian gene family in several respects. Considering the importance of *s100 *genes in the pathogenesis of epidermal disease – particular S100 proteins are markedly over expressed in psoriasis, wound healing, skin cancer, inflammation, cellular stress, and other epidermal pathological states [30] – it appears advantageous to study these processes in the zebrafish model system (*cf*. [48]).

## Methods

### Zebrafish strains and maintenance

All zebrafish used in this experiment were obtained from matings of the Ab/Tü strain (Oregon, AB/Tübingen, Tü). Breeding and raising of zebrafish followed standard protocols [49].

### Data mining

All annotated fish and mammalian S100 sequences were extracted either from the National Center for Biotechnology Information (NCBI) or Ensembl database resources [50] and used in the subsequent phylogenetic analysis, if they conformed to the inclusion criteria detailed below. Representative fish genes from each subclade and additionally, 19 human S100 genes (full set excluding close relatives [38]) were used as query in tBLASTN searches in the NCBI nucleotide databases nr/nt, EST [51] and in the ENSEMBL database [50]. For elephant shark (*Callorhinchus milii*) only WGS traces were available, and for salmon (*Salmo salar*) only the EST database was used. The analysis was repeated using newly found subclades as query. To be considered as validated S100 genes, the candidates needed to fulfill the following inclusion criteria: a) position within the S100 clade in the phylogenetic analysis; b) application of the BLASTP algorithm in the NCBI nonredundant database should result in annotated S100 or some other S100 candidates as first hits; c) presence of four helices separated by one S100 EF hand, one hinge and one canonical S100 hand (region assignment according to [1]) within 80 to 100 consecutive amino acids, which is the extent observed for this composite motif for all mouse and human S100 genes.

For Accession numbers see Additional File 2.

### Phylogenetic analysis

MAFFT, version 5.8 [52], was employed for multiple protein alignments using the *E-INS-i *strategy with the default parameters. To estimate the phylogenetic relationships of the sequences we performed distance-based, maximum parsimony, and maximum likelihood analyses using the Neighbour Joining (NJ), Protpars (MP) and Proml (ML) programs as implemented in ClustalX [53] and PHYLIP [22], packages respectively. For the NJ method we performed bootstrapping with 10000 repetitions using ClustalX [53] and for the MP and ML methods we performed bootstrapping with 100 repetitions using the program SEQBOOT from the PHYLIP package [22]. The three methods gave similar clustering. Consensus trees were obtained using the CONSENSE program of the PHYLIP package [22].

Subclades within the teleost S100 gene family were determined from the tree as the largest clades that fulfilled two criteria: the clade had >80% bootstrap support in the NJ analysis (S100Q is the only exception with 71%) and is supported both in the MP and ML analysis. Fourteen such subclades were identified, which correspond to groups of orthologous genes.

### Evolutionary distances

The evolutionary distances between amino acid sequences were calculated using MEGA4 [54]. All results were based on the pairwise analysis of the given number of sequences per ortholog or paralog group. Analyses were conducted using the Poisson correction method in MEGA4 [54,55]. All positions containing alignment gaps and missing data were eliminated only in pairwise sequence comparisons (Pairwise deletion option).

### Sequence logos

Sequence logos were generated using a web-based program, Weblogo, version 2.8.2. developed by Crooks [56] and Schneider and Stevens [57,58]. A logo was generated with 85 teleost and cartilaginous fish S100 amino acid sequences. Sequence alignments were manually edited using MEGA 4 [54] and highly divergent pieces between the start codon and the beginning of helix 1 were trimmed to avoid N-terminal length heterogeneity. This did not affect significantly conserved residues. Gap positions present in more than 85% of the sequences were deleted completely.

### dN/dS Analysis

The global dN/dS ratios for the full length S100 coding sequences of all five teleost species for which full genomic information is available were determined using the HyPhy package on the datamonkey server [59], which implements a previously published method [60]. The nucleotide alignment was manually edited and gap positions present in more than 85% of the sequences were removed.

To make inferences about selective pressure (positive and negative selection) on individual codons (sites) within the S100 coding sequences, the Single Likelihood Ancestor Counting (SLAC) package [61] was used, which implements the Suzuki-Gojobori method [60].

The algorithm is briefly outlined. First, a best-fitting nucleotide substitution model was automatically selected by fitting several such substitution models to both the data and a neighbor-joining tree generated from the alignment described above. Taking the obtained substitution rates and branch lengths as constant, a codon model was employed to fit to the data and a global dN/dS ratio was calculated. Then a codon by codon reconstruction of the ancestral sequences was performed using maximum likelihood. Afterwards the expected normalized (ES) and observed numbers (EN) of synonymous (NS) and non-synonymous (NN) substitutions were calculated for each non-constant site. dN = NN/EN and dS = NS/ES were then computed, and if dN < dS (negative selection) or dN > dS (positive selection), a p-value derived from a two-tailed extended binomial distribution was used to assess significance. Tests on simulated data (S.L.K. Pond and S.D.W. Frost, methods available at [61] show that p values equal or smaller than 0.1 identify nearly all true positives with a false positive rate generally below the nominal p value; for actual data, the number of true positives at a given false positive rate is lower. In the present study, two thresholds for significance (0.1 and 0.2) were taken into account.

### RT-PCR

Ten zebrafish (mix of male and female *Danio rerio*, strain Ab/Tü) were dissected and several tissues were pooled for each RNA extraction: barbels and lips, bone, brain, eyes, genitourinary, gills, heart, liver, muscle, olfactory bulb, olfactory epithelium, skin. cDNA was generated by using Superscript III reverse transcriptase (Invitrogen) with an anchored oligo18(dT) reverse primer. PCR amplifications were performed by using the following primer pairs, all of them (except S100B) intron-spanning:

*Dr_ *actin (forward, CCCCATTGAGCACGGTATT; reverse, TCATGGAAGTCCACATGGCAGAAG), *Dr *S100A1 (forward, CTTCAAGGGGAACTCAGTGA; reverse, AAAACTCATTGCATGCCACA), *Dr *S100A10b (forward, CGCAGGACATTCACATCATT; reverse, TTTTCCCCTCATGTTTGGTC), *Dr *S100A10a (forward, ATTTCACTCAGTCGCCCAAA; reverse, ATGGACAAACCCAAGACCAA), *Dr *S100A11 (forward, TCAAGGCTTATGCTGGGAAG; reverse, TGCAACATTGCCAATCAGA), *Dr *S100B (forward, GAAAGTTTGGACACCGATGG; reverse, TGGCCATGTCTTGAAACAAA), *Dr *S100I.1 (forward, AGAACCACCATGGCTACGTC; reverse, TGCAAAGCATTGTGATACAGG), *Dr *S100I.2 (forward, TCATTGCAACCTTCCACAAA; reverse, ACAGGCGATCAATGTGATGT), *Dr *S100S (forward, TGCAGATGCTCATCAAGACC; reverse, GTCCAGGAAGAAGTCGTTGC), *Dr *S100T (forward, TGGGAATGAGGGTGACAAAT; reverse, TCATTCGCTGGTCATGTGTT), *Dr *S100Z (forward, TAAACTGGAGGGAGCAATGG; reverse, TCCAGCACTCAGTTTACGAT). The following conditions were used: 2 min at 96°C, followed by 35 cycles of 30 sec at 96°C, 30 sec at 60°C, and 60 sec at 72°C, and a final extension of 10 min at 72°C. Regions chosen for PCR primers did not exhibit any appreciable sequence identity to each other (with exception of I2 primers, which may additionally recognize I1, but not vice versa), thereby excluding cross-amplification. All PCR products were cloned and sequenced using standard protocols. For sequences see Additional File 6.

### *In Situ *Hybridization

The templates for the probes were amplified from cloned fragments obtained by RT-PCR using the previously described primers with the T3 promoter site (TATTAACCCTCACTAAAGGGAA) attached to their 5' end. Digoxigenin (DIG) probes were synthesized according to the DIG RNA labeling kit supplier protocol (Roche Molecular Biochemicals).

RNA *in situ *hybridization of S100 genes was carried out following the method of Thisse et al [62] as modified in [63]. Hybridizations were performed on 5 dpf old larvae overnight at 62°C. Anti-DIG primary antibody coupled to alkaline phosphatase (Roche Molecular Biochemicals) and NBT-BCIP (Roche Molecular Biochemicals) were used for signal detection. Results were documented with a Nikon CoolPix 950 digital camera attached to a Nikon SMZ-U binocular for whole mount images. Cryosections of hybridized embryos, obtained by a Leica CM1900 cryostat were documented on a Zeiss AxioVert microscope and an attached Diagnostic Instruments Spot-RT camera.

## Authors' contributions

AK carried out the data mining, performed the sequence alignment, participated in the bioinformatic analysis, carried out the molecular cloning and the in situ hybridisation studies including photographic documentation, participated in the design of the study and drafted the Introduction. LS participated in the design of the study, the data mining, the sequence alignment and the bioinformatic analysis, and drafted the Methods section of the Manuscript. SK conceived of and designed the study, participated in the data mining and the bioinformatic analysis and wrote the manuscript. All authors read and approved the final manuscript.

## Supplementary Material

Additional File 1 **Phylogenetic tree of the S100 genes**. The cladogram represented here corresponds to the unrooted tree in Figure 1A. Red lines represent fish *s100 *genes, orange lines represent cartilaginous fish *s100 *genes, green lines represent lamprey *s100 *genes, light-blue represents human *s100 *genes (full set excluding close relatives, see [38]), and black represents the outgroup (troponins). The colored names indicate fish and lamprey *s100 *genes previously published. The Tn_S100T fragment (2nd exon) was not included for technical reasons. Click here for file

Additional File 2 **S100 fish genes**. Nomenclature, accession numbers, position and genomic location are shown. Genomic location was retrieved from the Ensembl database, release 47 [50], based on assembly zv7, April 2007 for zebrafish, assembly Fugu 4.0, June 2005 for *Takifugu rubripes*, assembly HdrR, October 2005 for medaka, assembly BROAD S1, February 2006 for stickleback, and assembly TETRAODON 7, April 2003 for *Tetraodon nigroviridis*. Click here for file

Additional File 3 **Evolutionary distances between S100 genes**. Mean evolutionary distances within both ortholog and paralog groups of the fish *s100 *genes. Note that orthologs genes of the following species were included in this analysis: *Danio rerio, Gasterosteus aculeatus, Oryzias latipes, Tetraodon nigroviridis *and *Takifugu rubripes*. Click here for file

Additional File 4 **Amino acid alignment of all the fish S100 genes**. The alignment is the same that was used for the construction of the sequence logo and it was manually edited (see Methods section). * Three genes in subfamily S100U share a C-terminal extension of 130–140 amino acids, which is abridged here. Click here for file

Additional File 5 **Number of positive and negative selected sites resulting from the site-by-site dN/dS analysisDescription: **Analysis was performed as described in Materials and Methods. Results for fourteen ortholog groups are shown. Click here for file

Additional File 6 **Nucleotide sequences of the cloned S100 cDNA fragments**. Fragments were generated by RT-PCR and cloned as described in Materials and Methods. Predicted exons are visualized by alternating blue and black color. Click here for file
