# Supplementary figures and images for: Structural and functional diversification in the teleost S100 family of calcium-binding proteins
Source: BMC Evol Biol. 2008 Feb 14;8:48. doi: 10.1186/1471-2148-8-48 (PMC2266712; doi:10.1186/1471-2148-8-48)

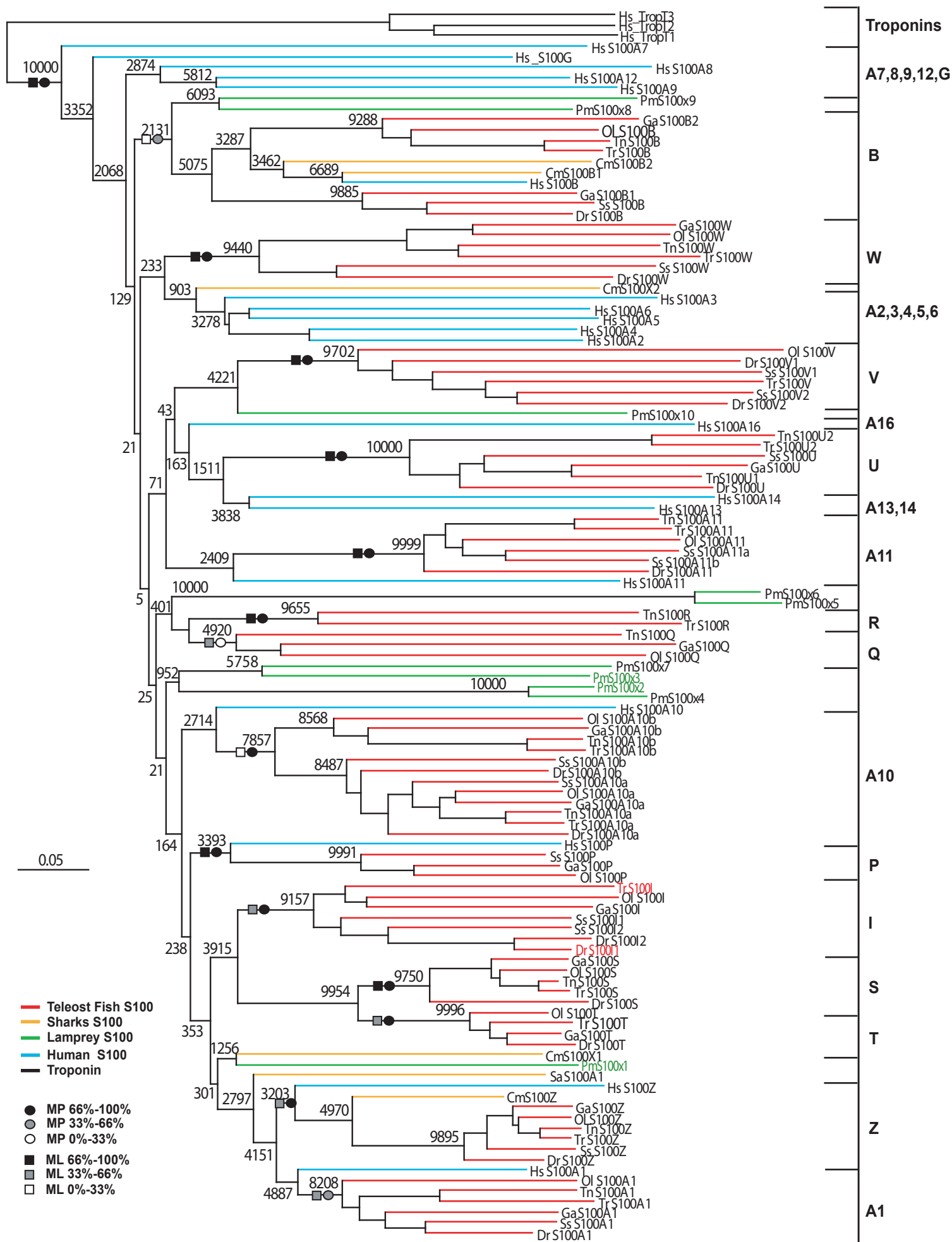

Supplement: Additional File 1 — Phylogenetic tree of the S100 genes. The cladogram represented here corresponds to the unrooted tree in Figure 1A. Red lines represent fish s100 genes, orange lines represent cartilaginous fish s100 genes, green lines represent lamprey s100 genes, light-blue represents human s100 genes (full set excluding close relatives, see [38]), and black represents the outgroup (troponins). The colored names indicate fish and lamprey s100 genes previously published. The Tn_S100T fragment (2nd exon) was not included for technical reasons. [file 1471-2148-8-48-S1.pdf]

## S100 Sequence Alignment

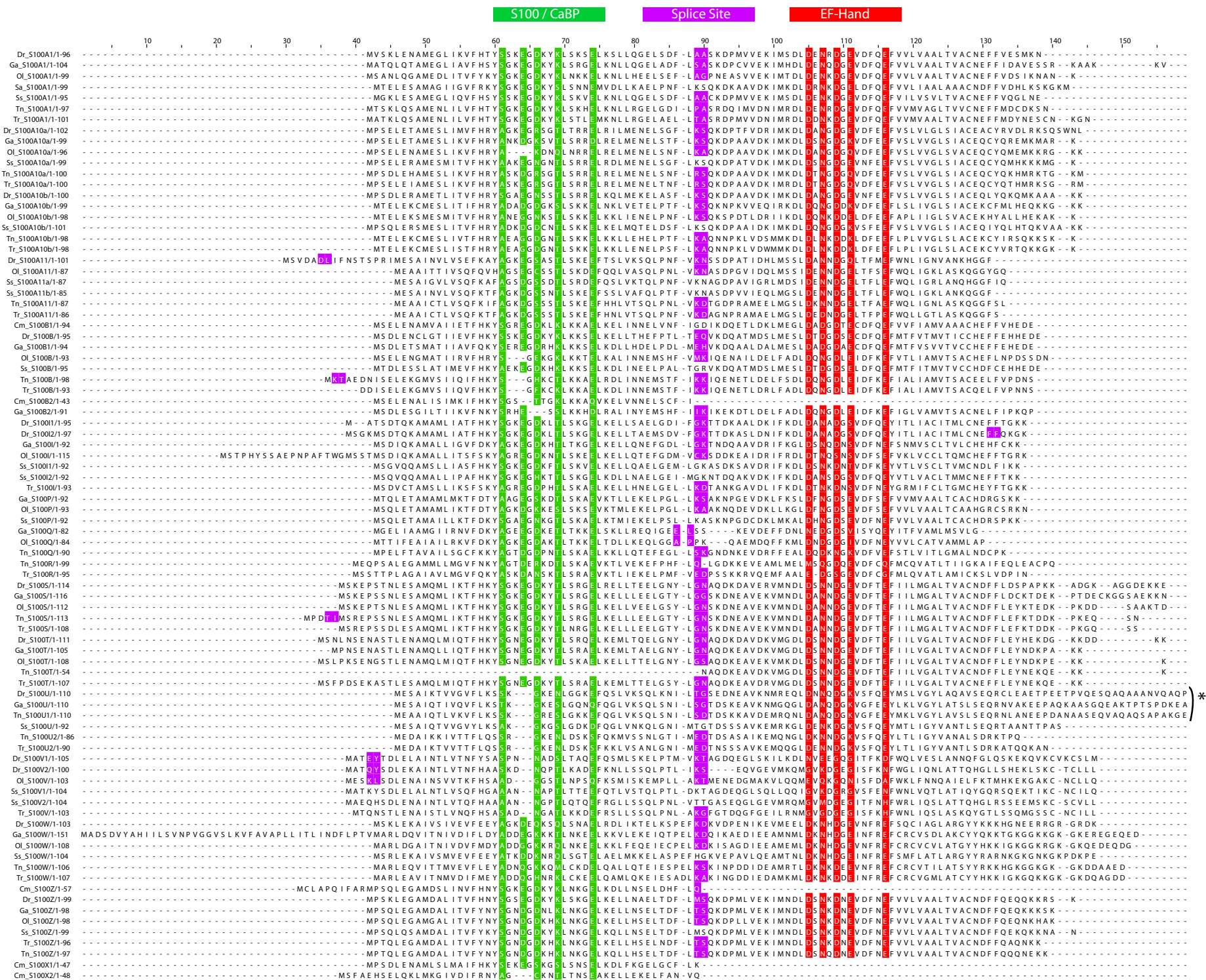

\* Sequences cut for clarity

Supplement: Additional File 4 — Amino acid alignment of all the fish S100 genes. The alignment is the same that was used for the construction of the sequence logo and it was manually edited (see Methods section). * Three genes in subfamily S100U share a C-terminal extension of 130–140 amino acids, which is abridged here. [file 1471-2148-8-48-S4.pdf]
