# Supplementary material for: Structural and functional diversification in the teleost S100 family of calcium-binding proteins
Source: BMC Evol Biol. 2008 Feb 14;8:48. doi: 10.1186/1471-2148-8-48 (PMC2266712; doi:10.1186/1471-2148-8-48)
Supplement: Additional File 2 — S100 fish genes. Nomenclature, accession numbers, position and genomic location are shown. Genomic location was retrieved from the Ensembl database, release 47 [50], based on assembly zv7, April 2007 for zebrafish, assembly Fugu 4.0, June 2005 for Takifugu rubripes, assembly HdrR, October 2005 for medaka, assembly BROAD S1, February 2006 for stickleback, and assembly TETRAODON 7, April 2003 for Tetraodon nigroviridis. [file 1471-2148-8-48-S2.pdf]

Organism	Gene	Accession No. GenBank [Ensembl.org]	Definition, former names or source	Chr. Location*
Callorhinchus milii	s100b1	AAVX01009655	WGS: 3311..3174, 1898..1755	n/a
Callorhinchus milii	s100b2	AAVX01078029	WGS: 1035..907	n/a
Callorhinchus milii	s100x1	AAVX01037989.1	WGS: 2008..2148	n/a
Callorhinchus milii	s100x2	AAVX01611743	WGS: 7..150	n/a
Callorhinchus milii	s100z	AAVX01332763	WGS: 511..341	n/a
Danio rerio	s100a1	NP_001082820	s100a1	(16) 20,379,814-20,380,186
Danio rerio	s100a10a	XP_001341092.1	unnamed hypothetical protein	(19) 6,293,004-6,293,712
Danio rerio	s100a10b	NP_998168.1	unnamed hypothetical protein	(16) 23,827,048-23,828,355
Danio rerio	s100a11	XP_001340375.1	hypothetical protein	(19) 5,252,567-5,254,627
Danio rerio	s100b	XP_001337819.1	similar to S100 calcium binding protein, beta (neural)	(22) 12,963,402-12,966,349
Danio rerio	s100i1	NP_997926.1	ictacalcin	(16) 23,859,904-23,861,147
Danio rerio	s100i2	NP_001076426.1	hypothetical protein	(16) 23,915,770-23,919,936
Danio rerio	s100s	EH439436	EST: 121...465	(19) 5,289,103-5,292,873
Danio rerio	s100t	EH472059	EST: 328 .. 663	(16) 23,181,892-23,182,313
Danio rerio	s100u	EV762118	EST: 83-763	(19) 5,259,713-5,273,865
Danio rerio	s100v1	XP_684054	unnamed hypothetical protein	(16) 21,026,052-21,030,978.
Danio rerio	s100v2	XP_001342023	unnamed hypothetical protein	(19) 14,992,814-14,995,932
Danio rerio	s100w	XP_698471.1	hypothetical protein	(16) 23,832,831-23,833,679.
Danio rerio	s100z	XP_001332692.1	unnamed hypothetical protein	n/a (21) v6
Gasterosteus aculeatus	s100a1	DW676049.1	EST 41..355	scaffold_122
Gasterosteus aculeatus	s100a10a	DT962380	EST: 9-308	groupX
Gasterosteus aculeatus	s100a10b	DW676026	EST: 53 .. 352	groupXX
Gasterosteus aculeatus	s100b1	DV013372	EST 62..346	groupI
Gasterosteus aculeatus	s100b2	AANH01007677	WGS: 20365-20237; 19642-19496	scaffold_27
Gasterosteus aculeatus	s100i	DN720770	EST: 31 .. 309	groupX

Organism	Gene	Accession No. GenBank [Ensembl.org]	Definition, former names or source	Chr. Location*
Gasterosteus aculeatus	s100p	bt027396	EST: 49 .. 327	groupVII
Gasterosteus aculeatus	s100q	AANH01001172	WGS: 24675-24544; 24434-24374	n/a
Gasterosteus aculeatus	s100s	AANH01002267	WGS: 191984-191831; 191628-191429	groupX
Gasterosteus aculeatus	s100t	AANH01001183; [ENSGACP00000016004]	WGS: 54126-54281; 54373-54535	groupXX
Gasterosteus aculeatus	s100u	DW034585	EST: 252 .. 938	groupX
Gasterosteus aculeatus	s100w	AANH01001172	WGS: 34320-34053; 33937-33749	n/a
Gasterosteus aculeatus	s100z	AANH01004842	WGS: 27004-26864; 25495-25332	groupXIV
Oryzias latipes	s100a1	BAAF04018396; [ENSORLP00000019653]	WGS: 727-868; 975-1133	(16) 24,358,752-24,359,158
Oryzias latipes	s100a10a	DC247185	EST: 57 .. 347	(11) 686,761-687,124
Oryzias latipes	s100a10b	DC246037	EST: 38-334	(16) 15,475,110-15,475,510
Oryzias latipes	s100a11	AM144689	EST: 113-376	n/a
Oryzias latipes	s100b	BAAF04004369; [ENSORLP00000019509]	WGS: 1193-1065; 797-644	(5) 28,377,654-28,378,197
Oryzias latipes	s100i	AM155215	EST: 78..425	(11) 690,546-691,440
Oryzias latipes	s100p	AM156198	EST: 96-377	(18) 26,574,093-26,574,750
Oryzias latipes	s100q	AM140377	EST: 84-338	(16) 15,491,709-15,492,049
Oryzias latipes	s100s	BAAF04089124	WGS: 1557-1406; 1260-1074	(11) 366,322-366,778
Oryzias latipes	s100t	BJ877870	EST: 35-361	(16) 15,215,652-15,216,886
Oryzias latipes	s100v	DC253174	EST: 83-394	n/a
Oryzias latipes	s100w	DC268269	EST:109-435	(16) 15,482,948-15,483,345
Oryzias latipes	s100z	BAAF04000354; [ENSORLP00000002753]	WGS: 4532-4672; 6621-6780	(12) 2,541,103-2,543,351
Petromyzon marinus	s100x1	EC384676.1	see Ref. [14] (S100_Pma1)	n/a
Petromyzon marinus	s100x10	[GENSCAN00000118637]	n/a	n/a
Petromyzon marinus	s100x2	EB081469.1	see Ref. [14] (S100_Pma2)	n/a
Petromyzon marinus	s100x3	DY251122.1	see Ref. [14] (S100_Pma3)	n/a
Petromyzon marinus	s100x4	EC384389.1	EST: 83 .. 403	n/a
Petromyzon marinus	s100x5	EB391547	EST: 80 .. 406	n/a

Organism	Gene	Accession No. GenBank [Ensembl.org]	Definition, former names or source	Chr. Location*
Petromyzon marinus	s100x6	DY798632.1	EST: 93 .. 419	n/a
Petromyzon marinus	s100x7	[GENSCAN00000111444]	n/a	n/a
Petromyzon marinus	s100x8	[GENSCAN00000006715]	n/a	n/a
Petromyzon marinus	s100x9	EB392838.1	EST: 24 .. 386	n/a
Salmo salar	s100a1	EG803197	EST: 55 .. 345	n/a
Salmo salar	s100a10a	EG931150	EST: 60 .. 359	n/a
Salmo salar	s100a10b	DW471500	EST: 66..371	n/a
Salmo salar	s100a11a	EG927362	EST: 77..340	n/a
Salmo salar	s100a11b	EG875327	EST: 80..337	n/a
Salmo salar	s100b	EG811546	EST: 21 .. 317	n/a
Salmo salar	s100i1	EG775556	EST: 78 .. 356	n/a
Salmo salar	s100i2	EG801949	EST: 3 .. 344	n/a
Salmo salar	s100p	EG874901	EST: 87..365	n/a
Salmo salar	s100u	EG935648	EST:117..395	n/a
Salmo salar	s100v1	EG883856	EST: 48..362	n/a
Salmo salar	s100v2	CB513603	EST: 11..325	n/a
Salmo salar	s100w	EG786462.	EST: 81..395	n/a
Salmo salar	s100z	DW547467	EST: 51 .. 350	n/a
Squalus acanthias	s100a1	AAN63527	S-100 calcium-binding protein A1	n/a
Takifugu rubripes	s100a1	CAAB01000479; [SINFRUG00000127285]	WGS: 48572-49084; 48931-49084	(sc. 175) 184,238-184,719
Takifugu rubripes	s100a10a	CA844972	EST: 36-338	(sc. 37) 608,876-609,210
Takifugu rubripes	s100a10b	CAAB01000082; [SINFRUP00000145915]	WGS: 68471-68333; 68247-68089	(sc. 252) 290,924-291,300
Takifugu rubripes	s100a11	AL835704	EST: 3-246 (incomplete)	(sc. 37) 453,156-453,94
Takifugu rubripes	s100b	CAAB01000495; [SINFRUG00000136068]	WGS: 113500 - 113650; 114105-114253	(sc. 33) 965,137-965,890
Takifugu rubripes	s100i	AL835667	EST: 12 .. 293	(sc. 37) 613,191-613,571
Takifugu rubripes	s100r	CA846676	EST: 27-314	(sc. 164) 304,072-304,440

Organism	Gene	Accession No. GenBank [Ensembl.org]	Definition, former names or source	Chr. Location*
Takifugu rubripes	s100s	CAAB01002685, [SINFRUG00000141424]	WGS: 5679-5527; n/a	(sc. 37) 444,437-444,949
Takifugu rubripes	s100t	CAAB01003369	WGS: 15712-15551; 15464-15302	(sc. 252) 102,893-103,264
Takifugu rubripes	s100u1	CAAB01002685	WGS: 10538-10419; 10195-9623	(sc. 37) 448,942-449,677
Takifugu rubripes	s100u2	BU808303	EST: 129-401	(sc. 252) 105,484-105,827
Takifugu rubripes	s100v	BU808261	EST: 50 .. 361	(sc. 69) 1,175,453-1,176,463
Takifugu rubripes	s100w	CAAB01000082; [SINFRUP00000179345]	WGS: 62793-62652; 62508-62384	(sc. 252) 296,602-297,011
Takifugu rubripes	s100z	CAAB01009030; [SINFRUP00000136497]	WGS: 3430-3290; 2181-2031	(sc.27) 131,446-132,845
Tetraodon nigroviridis	s100a1	CAAE01007788	** WGS: 1634-1775; 2049-2201	n/a
Tetraodon nigroviridis	s100a10a	CAG10829	** WGS: 442617-442477; 442405-442243	21
Tetraodon nigroviridis	s100a10b	CAG11861	** WGS: 162056-161918; 161815-161657	8
Tetraodon nigroviridis	s100a11	CAG10836	** WGS: 616461-616589, 617105-617239	21
Tetraodon nigroviridis	s100b	CAF96543	** WGS: 1224964-1225109; 1225576-1225722	11
Tetraodon nigroviridis	s100q	CAG10828	** WGS: 439126-438989; 438902-438768	21
Tetraodon nigroviridis	s100r	CAAE01002256	** WGS: 1461-1607; 1706-1857	n/a
Tetraodon nigroviridis	s100s	CAG10837	** WGS: 625839-625852; 626074-626227; 626437-626611	21
Tetraodon nigroviridis	s100t	CAAE01015044	WGS: 328871...329035	8
Tetraodon nigroviridis	s100u1	CR728411	EST , 36-725	n/a
Tetraodon nigroviridis	s100u2	CR733746.2	EST: 123 .. 383	n/a
Tetraodon nigroviridis	s100w	CAG11860	WGS: 158279-158139; 158074-157891	8
Tetraodon nigroviridis	s100z	CAG05262	** WGS: 1357545-1357405; 1356444-1356291	n/a

* Based on the assemblies of ensembl.org (Zebrafish: Zv7, Apr 2007; Takifugu: FUGU 4.0, Jun 2005; Medaka: HdrR, Oct 2005; Tetraodon: TETRAODON 7, Apr 2003; Stickleback: BROAD S1, Feb 2006)
** unnamed protein product; automated annotation by Genescan
