# Supplementary material for: Structural and functional diversification in the teleost S100 family of calcium-binding proteins
Source: BMC Evol Biol. 2008 Feb 14;8:48. doi: 10.1186/1471-2148-8-48 (PMC2266712; doi:10.1186/1471-2148-8-48)
Supplement: Additional File 3 — Evolutionary distances between S100 genes. Mean evolutionary distances within both ortholog and paralog groups of the fish s100 genes. Note that orthologs genes of the following species were included in this analysis: Danio rerio, Gasterosteus aculeatus, Oryzias latipes, Tetraodon nigroviridis and Takifugu rubripes. [file 1471-2148-8-48-S3.pdf]

Mean evolutionary distance between ortholog and paralog amino acid sequence groups

	Orthologs															Paralogs					
	**A1**	**A10**	**A11**	**B**	**I**	**P**	**Q**	**R**	**S**	**T**	**U**	**V**	**W**	**Z**	**Avr.**	**Dr**	**Ol**	**Ga**	**Tn**	**Tr**	**Avr.**
Mean Evol. Distance	0,34	0,44	0,37	0,41	0,47	0,25	0,82	0,69	0,15	0,13	0,56	0,79	0,56	0,11	0,44	1,04	1,07	0,97	1,12	1,18	1,08
