# Supplementary material for: Structural and functional diversification in the teleost S100 family of calcium-binding proteins
Source: BMC Evol Biol. 2008 Feb 14;8:48. doi: 10.1186/1471-2148-8-48 (PMC2266712; doi:10.1186/1471-2148-8-48)
Supplement: Additional File 5 — Number of positive and negative selected sites resulting from the site-by-site dN/dS analysisDescription: Analysis was performed as described in Materials and Methods. Results for fourteen ortholog groups are shown. [file 1471-2148-8-48-S5.pdf]

**Number of positive and negative selected sites resulting from the site-by-site dN/dS analysis**

| | p<0.1 | | p<0.2 | |
|---|---|---|---|---|
| | **Positive Sites** | **Negative Sites** | **Positive Sites** | **Negative Sites** |
| **A1** | 0 | 14 | 0 | 39 |
| **A10** | 0 | 43 | 0 | 54 |
| **A11** | 0 | 8 | 0 | 23 |
| **B** | 0 | 13 | 0 | 30 |
| **I** | 0 | 11 | 0 | 24 |
| **P** | n.d. | n.d. | n.d. | n.d. |
| **Q** | 0 | 6 | 0 | 13 |
| **R** | n.d. | n.d. | n.d. | n.d. |
| **S** | 0 | 15 | 0 | 28 |
| **T** | 0 | 19 | 0 | 45 |
| **U** | 0 | 10 | 0 | 23 |
| **V** | 0 | 10 | 0 | 26 |
| **W** | 0 | 19 | 0 | 34 |
| **Z** | 0 | 17 | 0 | 34 |
| Average | 0,0 | 15,4 | 0,0 | 31,1 |
