# Supplementary material for: Structural and functional diversification in the teleost S100 family of calcium-binding proteins
Source: BMC Evol Biol. 2008 Feb 14;8:48. doi: 10.1186/1471-2148-8-48 (PMC2266712; doi:10.1186/1471-2148-8-48)
Supplement: Additional File 6 — Nucleotide sequences of the cloned S100 cDNA fragments. Fragments were generated by RT-PCR and cloned as described in Materials and Methods. Predicted exons are visualized by alternating blue and black color. [file 1471-2148-8-48-S6.pdf]

S100A1

CTTCAAGGGGAACTCAGTGACTTTTTAGC
CGCAAGTAAAGACCCCATGGTGGTGGAGAAGATCATGTCTGATCTGGATG
AGAACCGGGATGGAGAGGTGGACTTTCAGGAGTTTGTTGTGCTGGTGGCT
GCTCTCACTGTGGCATGCAATGAGTTTTAATCTGAATTCGTCGACAAGCT

S100A10b

TCGCAGGACATTCACATCATTCCTTCATCTT
CAGGTGACGCTCTGCCTTTATCAGCACATTCACAATGCCATCTGATCTGG
AGAGAGCAATGGAGACCCTGATCACGGTGTTTCACCGTTATTCAGGCGCA
GAGGGGAACTCATCAACCCTGAGTCGAAGAGAGCTTAAACAGCTGATGGA
GAAAGAGCTGGCTAGTTTTCTGAAGAGTCAGAAAGACCCTGCTGCCGTAG
ACAAGATTATGAAGGATCTGGATGCCAATGGGGATGGAGAGGTGAATTTT
GAGGAGTTTGTGTCTCTTGTGGTGGGCCTGTCCATCGCCTGCGAACAACT
CTACCAGAAACAGATGAAAGCAGCAGCCAAGAAGTAATAAAAGACCAAAC
ATGAGGGGAAAAATCTGAATTCGTCGACAAGCTTCTCGAGCCTAGGCTAG

S100A10a

TA
TTTCACTCAGTCGCCCAAACGTGCTTGACCTGAGCATCGCGCAGCCATGC
CATCCGAACTAGAGACCGCCATGGAGTCCCTCATCATGGTTTTCCACCGA
TATGCTGGAAAGGAGGGCAGGAGCGGCACCCTGACCCGACGGGAACTCCG
AATACTAATGGAGAACGAGCTCTCTGGGTTCCTTAAGTCTCAGAAGGATC
CGACATTCGTCGACAGAATTATGAAAGATCTGGATGCCAATGGAGACGGC
GAGGTTGACTTTGAGGAGTTTGTGTCTTTGGTCTTGGGTTTGTCCATAAT

S100A11

TCANCCCTTATGCTGGGAAGGAAGGTTCAGCAAGTACTCTGA
GTAAAGAAGAGTTTACAAGCTTGGTCAAATCCCAGTTACCAAACTTTGTA
AAGAATTCATCTGATCCAGCCACCATTGACCATCTCATGAGCTCATTGGA
TGCGAATAATGATGGGCAGCTGACGTTCATGGAGTTCTGGAATCTGATTG
GCAATGTTGCA

S100B

TGAAAGTTTGGACA
CCGATGGCGACTCGGAGTGTGATTTCCAGGAGTTCATGACCTTCGTCACC
ATGGTTACCATCTGTTGCCATGAGTTTTTCGAACACCATGAAGACGAATG
AGTCTAAATAAAAGAAAGTAGTTTTCGTAGTTTAATGAGTCTCAATACAT
TTCTTCAGTAAACTTTACTTCGTTCTCATCGCATTAGGATTGTTTTATTG
TTGTTGTTTGTTTGGACAGAATGTACATGCAGCTTGTCTGACAATTTAGC
TTATTTCTGCTAGACTCGAAAATCAGTTCGTAATTTATTACAAAGCATGC
TTCTATTATTTTGGCTAGACATTTGTTTCAAGACATGGCCA

S100I1

TCA
NCGCTACCTTCCACAAATACTCTGGAAAGGAGGGCGACAAATTGACCCTG
TCCAAAGGCGAGCTGAAGGAACTGCTCTCTGCAGAGTTGGGCGACATCTT
TGGGAAAACTACAGACAAGGCAGCTTTGGACAAGATATTCAAGGATCTGG
ATGCAAATGCTGATGGTTCTGTGGACTTTCAGGAGTACATCACATTGATC
GCCTGT

S100S

TGCCCCTTTTCATCAAGACCTTTCATAAGTACTTGGGGAAGGAAGGAGAC
AAGTATACGCTCAGCCGAGGAGAACTGAGGGAACTTCTGACAGAGGAGCT
GGGGAATTATCTTGGGAACGCTCAAGATAAAGACGCGGTGGAGCGAGTGA
TGAACGATCTGGACTCCAATAACGACGGCGAGGTGGATTTCACCGAGTTC
ATCATCCTCATGGGCGCTCTGACCGTGGCCTGCAACGACTTCTTCCTGGA
C

S100T

TGGGAATGAGGGTGACAAATATACTCTCAGCAGACAGGAACTCAAAGAGA
TGTTAACGCAGGAGCTGGGAAACTACCTTGGGGTGAAGAGAGATTTAAAA
AAAAAAGAGTTTGCTTAGAGAAAGTATAAGATTCTGATTAACTTCATCTG
TATCTCCCTTACTCCACAGAATGCACAGGATAAGGATGCGGTAGATAAAG
TTATGGGAGATCTAGATTCAAACAACGATGGTGAGGTGGACTTCACAGAG
TTCATCATCCTCGTAGGTGCCCTCACCGTCGCCTGCAACGACTTCTTCCT
CGAGTATCATGAAAAGGATGGAAAGAAGGATGACAAGAAATAGAGACGCA
TTTGTCTAAAGCCAACACATGACCAGCGAATGA

S100Z

TAAACTGG
GGGAGCAATGGATGCGCTGATCACCGTCTTCCACAACTACTCCGGCAGT
ZGAAGGAGACAAATACAAACTGAGCAAAGGAGAGCTGAAGGAGCTTCTCAA
CGCTGAACTCACCGACTTCCTCATGTCTCAGAAGGACCCGATGCTGGTGG
AGAAGATCATGAACGATCTGGACTCCAACAAGGACAACGAGGTGGACTTT
AATGAGTTTGTGGTTCTGGTGGCGGCGCTGACTGTGGCCTGTAACGACTT
CTTCCAGGAACAACAGAAGAAAAGGAGCAAATAGAACACATTGTATACCC
GTAGTGCTCTATGACGCATTAAACCATCACAGCAGTCCTCCTGTTACTCC
ACATTCATCTACATCTTTGTATTTAAAATATTTAAAATGACAGTTTTCTC
ACATTAATGTATTTATCGTAAACTGAGTGCTGGA
